# Nitrogen–nitrogen bond-containing natural products: isolation, bioactivity, synthesis, biosynthesis, and pharmacology

**DOI:** 10.1007/s13659-026-00653-9

**Published:** 2026-11-11

**Authors:** Ziyi Wu, Mengjing Cong, Weimao Zhong, Yonghong Liu, Junfeng Wang

**Affiliations:** 1 Guangdong Key Laboratory of Marine Materia Medica, State Key Laboratory of Tropical Oceanography, South China Sea Institute of Oceanology, Chinese Academy of Sciences, Guangzhou, 510301 China; 2 University of Chinese Academy of Sciences, 19 Yuquan Road, Beijing, 100049 China; 3 Sanya Institute of Ocean Eco-Environmental Engineering, Sanya, 572000 China

**Keywords:** Nitrogen–nitrogen bond, Synthesis and biosynthesis, Pharmacology

## Abstract

Natural products that contain nitrogen-nitrogen (N–N) bonds are a class of natural products consisting of multiple functional groups with important biological activities. Currently, such molecules have been widely isolated from microorganisms, plants, and marine organisms. In recent years, due to the novel skeletal structures and unique biological activities of these compounds, research into their synthetic routes and mechanisms of action continues to be increasingly in-depth. Therefore, this review summarizes the isolation and identification of 131 N–N bond-containing compounds of microbial origin, biosynthesis and synthesis, with special emphasis on the summary of the mechanism of N–N bond formation and pharmacological elaboration during the period of 2014–2024, laying the foundation for future research into the drug development potential of compounds containing N–N bonds.

**Graphical abstract:**

**Figure:**
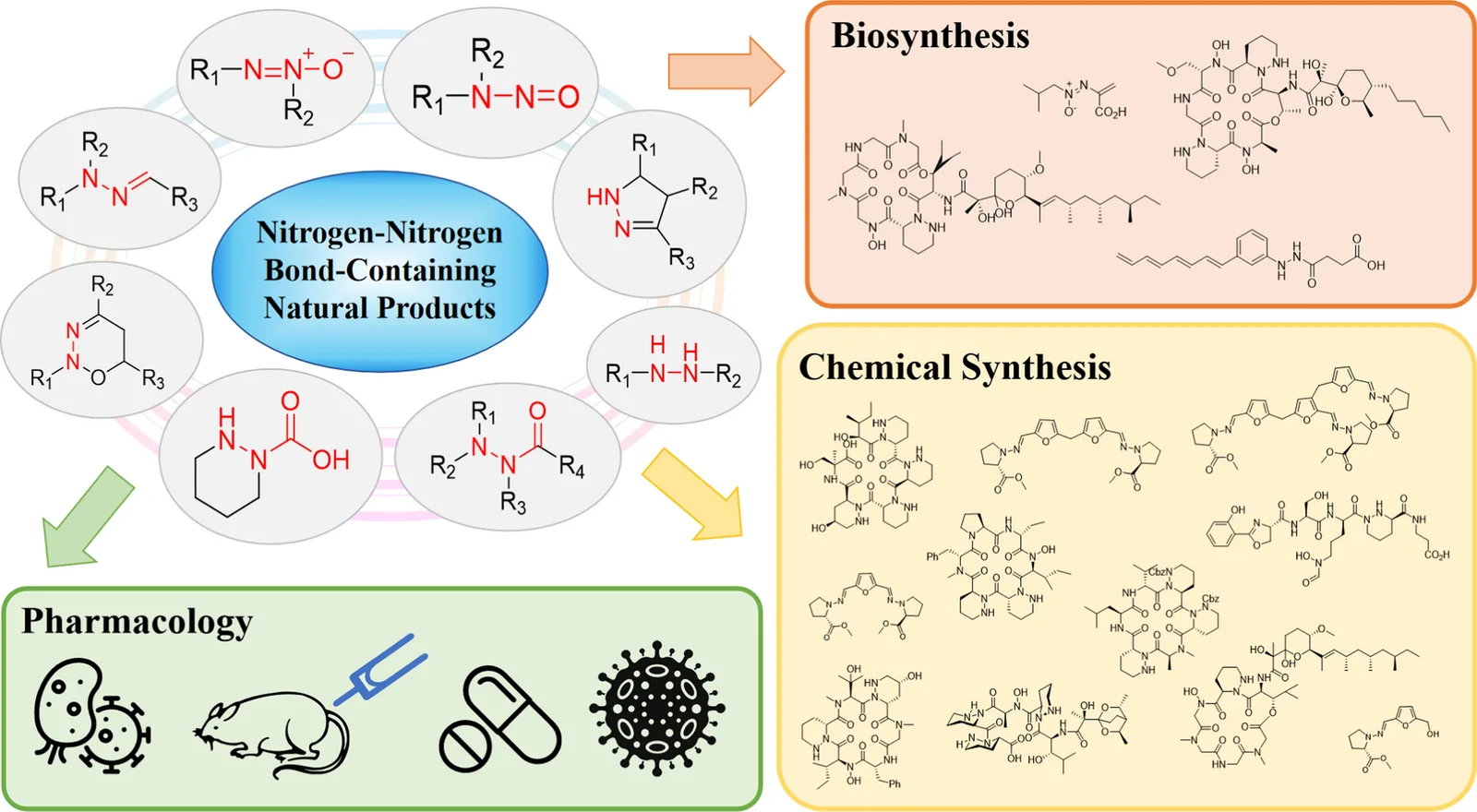

## Introduction

The diversity of natural products of microbial origin are widely distributed in different biological environments, such as terrestrial soils and aquatic environment [1], in particular the marine environment. That marine microorganisms provide sources for finding new biologically active natural products, including new drugs discovery area [2]. Since the first natural product macrozamin containing an nitrogen–nitrogen (N–N) bond was isolated and characterized in [3], more than 300 natural products with N–N bonds have been identified [4,5].

Compounds containing N–N bonds have a unique molecular structure and form a series of biologically active functional groups, including azoxy, azo, diazo, nitrosamines, nitroamines, nitrosohydroxylamines, hydrazides, hydrazones, hydrazines, azines, pyrazoles, indazoles and other heterocyclic structures [4], which occupy an irreplaceable position in synthetic chemistry and drug discovery.

First, these molecules display different structures and biological activities rarely observed in ordinary metabolites [6], such as anti-tumor, anti-inflammatory, anti-bacterial etc. [7]. For instance, lenziamides, whose unique hydrazine pharmacophore-containing peptide backbone confer a rigid cyclic conformation and enable highly selective antiproliferative activity against colon cancer cells, representing promising lead compounds for chemotherapy agents [8]. Secondly, many iron-binding compounds can serve as potential biological control agents against harmful plant pathogens and have the ability to mitigate heavy metal pollution, playing an important role in both ecosystem balance and the medical field [9]. For example, the diazeniumdiolate-type N–N metabolite gramibactin act as high-affinity iron carriers, promoting photosynthesis in a variety of plants and increase the yields by enhancing iron uptake [10]. Thirdly, there is a series of enzyme-catalyzed N–N bond formations that are rarely observed in other biosynthetic pathways. The heme-dependent piperazine synthase KtzT provides a novel biocatalytic tool for constructing nitrogen-containing heterocycles of the piperazine class and holds great potential in the field of green chemistry [11]. Collectively, the novel skeletal features, diverse biological activities and functions, and unique enzymatic mechanisms make N–N bond-containing natural products worthy of more systematic characterization to provide guidance for subsequent in-depth mechanistic studies.

Although hundreds of compounds have been isolated, the mechanisms involved in N–N bonds formation have only recently begun to emerging [12], including a detailed description of the mechanism in related reviews by researchers [6,13–17]. In 2017, Balskus et al. elaborated on enzymes catalyzing the formation of N–N bond in six classes of compounds containing N–N bond-containing functional groups [13]. In 2017, Guo and Lu [14] classified the N–N bond formation according to the intramolecular and intermolecular reactions. In 2020, Matsuda et al. focused on the biosynthetic pathways for several important classes of compounds such as hydrazine [15]. In 2021, Du et al. described the mechanism of hydrazine synthase in hydrazine formation [6]. In 2021, Zhao et al. classified the reactions of N–N bond formation into comproportionation, rearrangement, and radical recombination reactions [16]. In 2022, Du et al. provided a detailed description of the biosynthetic and chemical synthesis pathways of natural compounds containing N–N bond [17].

Two representative reviews on natural products containing N–N bonds have been published previously. Blair et al. [4] summarized only those natural products containing N–N bonds discovered prior to 2013. Le Goff et al. [5] focused exclusively on hydrazine-containing natural products, neglecting the biosynthesis, total chemical synthesis, or in-depth pharmacological mechanisms of other N–N frameworks, such as azoxides and piperazine acids. Unlike previous reviews, this study systematically compiles 131 newly isolated microbial N–N natural products reported between 2014 and 2024, adding some latest research results on biosynthesis and chemical synthesis, and analyzed the pharmacology of related compounds.

## Sources and biological activities of natural N–N compounds from microorganisms

### Innovative methods for isolation and structural characterization

With advances in science and technology in recent years, new separation techniques have gradually emerged in the field of natural product isolation that differ from traditional methods, such as simple column chromatography. This subsection systematically sorts mainstream modern technologies specialized for mining and identifying N–N bond-containing natural products, with typical research cases attached for each strategy.

#### Genome mining targeting N–N biosynthetic signature genes

Genome mining technology involves the targeted identification of genes encoding enzymes involved in the synthesis of secondary metabolites through biosynthetic gene clusters (BGCs). This process is carried out in conjunction with specialized analysis software (such as antiSMASH) to identify suitable target natural products, thereby reducing the randomness of the screening process to some extent [18]. Taking piperazine acid analogs as an example, through genome mining, high-throughput screening of a bacterial DNA library (2,020 strains) was performed using polymerase chain reaction (PCR) primer pairs based on conserved ktzI/ktzT homologous genes, thereby prioritizing the identification of potential producer strains and successfully identifying lenziamides and the depsidomycin D as novel N–N-type natural products [8].

#### ^15^N isotopic labeling combined with NMR spectroscopy

^15^N-NMR is a structural identification strategy based on the addition of ^15^N to the culture media of filamentous fungi and bacteria. Through novel two-dimensional NMR and mass spectrometry analysis strategies, this method directly localizes nitrogen atoms in pharmacophores that cannot be distinguished using ^1^H/^13^C NMR. There is a growing trend toward the identification of natural products using the ^15^N stable isotope feeding method [19]. For example, during the isolation and identification of incarnatapeptins, after screening candidate strains against the target genome, ^15^N NMR was employed as a specific identification tool. The N–H correlations of piperazine acid and its complete proton spin system were captured by applying the ^1^H-^15^N HSQC-TOCSY. At the same time, isotopically labeled ^15^N_2_-L-ornithine (a biosynthetic precursor of Piz) was added to the fermentation medium to enhance the signal, thereby enabling direct tracking of the target fraction via NMR-guided fractionation [20]. This spectral feature strategy has accelerated the discovery of dentigerumycins [21].

#### OSMAC culture strategy

“One Strain, Multiple Compounds” (OSMAC) highlights the mechanism by which a single microbial strain can produce different molecules when grown under varying environmental conditions. Therefore, by adjusting parameters such as nutrient concentration, temperature, and aeration rate, it is possible to alter the overall physiological state of a microbial strain, thereby significantly influencing the production of secondary metabolites. Compounds with UV absorption spectra significantly different from those of known compounds were identified using the OSMAC strategy, leading to the isolation and identification of talarohydrazones [22]. This method can effectively expand the chemical diversity of N–N metabolites.

#### GNPS molecular networking

GNPS is an open platform for the storage and analysis of MS/MS spectra. By comparing LC–MS/MS fragmentation patterns to construct a molecular network, metabolites with similar structures are grouped into distinct network nodes, thereby identifying target compound types [23]. Through GNPS, a series of lunaemycins were identified following high-throughput screening, representing a more precise approach for complex crude compounds containing N–N bonds, thereby eliminating the need for repetitive isolation work [24].

#### Co-cultivation strategy

Co-culture is a technique that can enhance the diversity of microbial metabolites [25]. This method allows microorganisms to exist in conditions closer to their natural habitats, increasing interspecific competition and chemical signaling, thereby activating silenced gene clusters in strains [26]. Numerous studies have now shown that bacterial-bacterial co-culture systems [27], fungal-fungal co-culture systems [28] can all activate the biosynthesis of natural products. However, traditional co-culture strategies also have some drawbacks. For example, it is difficult to identify the specific strain responsible for the induced production, and differences in the growth states of different strains may lead to poor experimental reproducibility [26]. For example, Kerr et al. used four heat-inactivated inducer strains to investigate their effects on the secondary metabolite profiles of seven actinomycete strains and successfully identified new natural products [26].

### Structural separation

#### Hydrazides

Incarnatapeptins A (**1**) and B (**2**) were isolated from *Streptomyces incarnatus* NRRL 8089 by genomic screening in 2020. In addition, 30 μM of incarnatapeptins A and B had no significant effect on PC3 cell density, but incarnatapeptin B was active and selectively sensitive to LNCaP human prostate cancer cells at 30 μM, whereas incarnatapeptin A was inactive [20].

Meliponamycin A (**3**) and meliponamycin B (**4**) were isolated from *Streptomyces* sp. ICBG1318 associated with *Melipona scutellaris* nurse bees in 2020. The absolute amino acid configuration was shown to be the L-configuration by Marfey’s analysis. The two compounds showed antimicrobial activity against the entomopathogen *Paenibacillus larvae* and *Staphylococcus aureus*, and high antiprotozoal activity against the protozoan parasite *Leishmania infantum* [29].

A new albofungin derivative, albofungin A (**5**), was isolated from *Streptomyces chrestomyceticus* in 2021. Compound **5** showed strong activity against both ESKAPE Gram-positive and Gram-negative bacteria and could present a dose-dependent induction of apoptosis in HeLa and MCF 7 cells. An antiSMASH analysis of the genome of the strain revealed a 72 kb gene cluster and suggested a biosynthetic pathway for albofungin [30].

Six new peptide homologs (**6**–**11**) of JBIR-39 and JBIR-40 containing piperazinic acid (PA) were isolated and characterized from endophytic *Streptomyces* sp. AB100. Of these six derivatives, only two dehydroxylated PA-containing hexapeptides exhibited antimicrobial activity against Bacillus subtilis, whereas the other four, probably linear pentapeptidic derivatives or degradation products of JBIR-39 or JBIR-40, had no similar antimicrobial activity [31].

Dentigerumycins F (**12**) and G (**13**) are new compounds isolated from *Streptomyces incarnatus* NRRL 8089 utilizing the ^15^N NMR guided method. When isolated in trifluoroacetic acid (TFA) containing HLPC eluents and stored in DMSO-*d*_6_, it exhibited the property of equilibrium between dehydrated and hydrated homologues, in addition to the property of tautomerization between a ketone and hemiketones [21].

Petrichorins A (**14**) and B (**15**) were identified from *Lentzea flaviverrucosa* DSM 44664 by the piperazate-targeted metabolic genomics united the genetics of piz monomer production and other methods. Petrichorin A is an asymmetric heterodimer: its left- and right-loop segments are assembled by NRPS machineries LtzDE and LtzUV, respectively. Cytochrome P450 LtzS catalyzed chloro-biaryl cyclopeptide cross-linking to produce this heterodimer. By contrast, petrichorin B is a homodimer generated from LtzDE-assembled precursors via LtzS-mediated cross-linking. In addition, petrichorins A and B showed growth inhibitory activity against four types of cancer cells including HT1080 fibrosarcoma, A2780 human ovarian cancer [32].

Polyoxyperuin A seco acid (**16**) was isolated by genome mining from *Streptomyces* sp. s120. Structural analysis showed that polyoxyperuin A could be hydrolyzed to yield the ring-opening product polyoxyperuin A seco acid. Bioactivity analysis revealed that polyoxyperuin A seco acid exhibited moderate activity against *Micrococcus luteus*. In addition, the researchers also proposed putative biosynthetic pathways for both compounds [33].

Lydiamycins E–H (**17**–**20**) were isolated from endophytic *Streptomyces* sp. HBQ95 in 2022. Among them, lydiamycins E is the second naturally occurring 13-membered piperazic acid-bearing depsipeptide. Lydiamycin H, lydiamycin G, and lydiamycin F were demonstrated to have migratory inhibitory activity against PANC-1 human pancreatic cancer cells. However, none of the lydiamycins E–H exhibited cytotoxicity against the PANC-1 pancreatic cancer cell line [34].

**Fig. 1:**
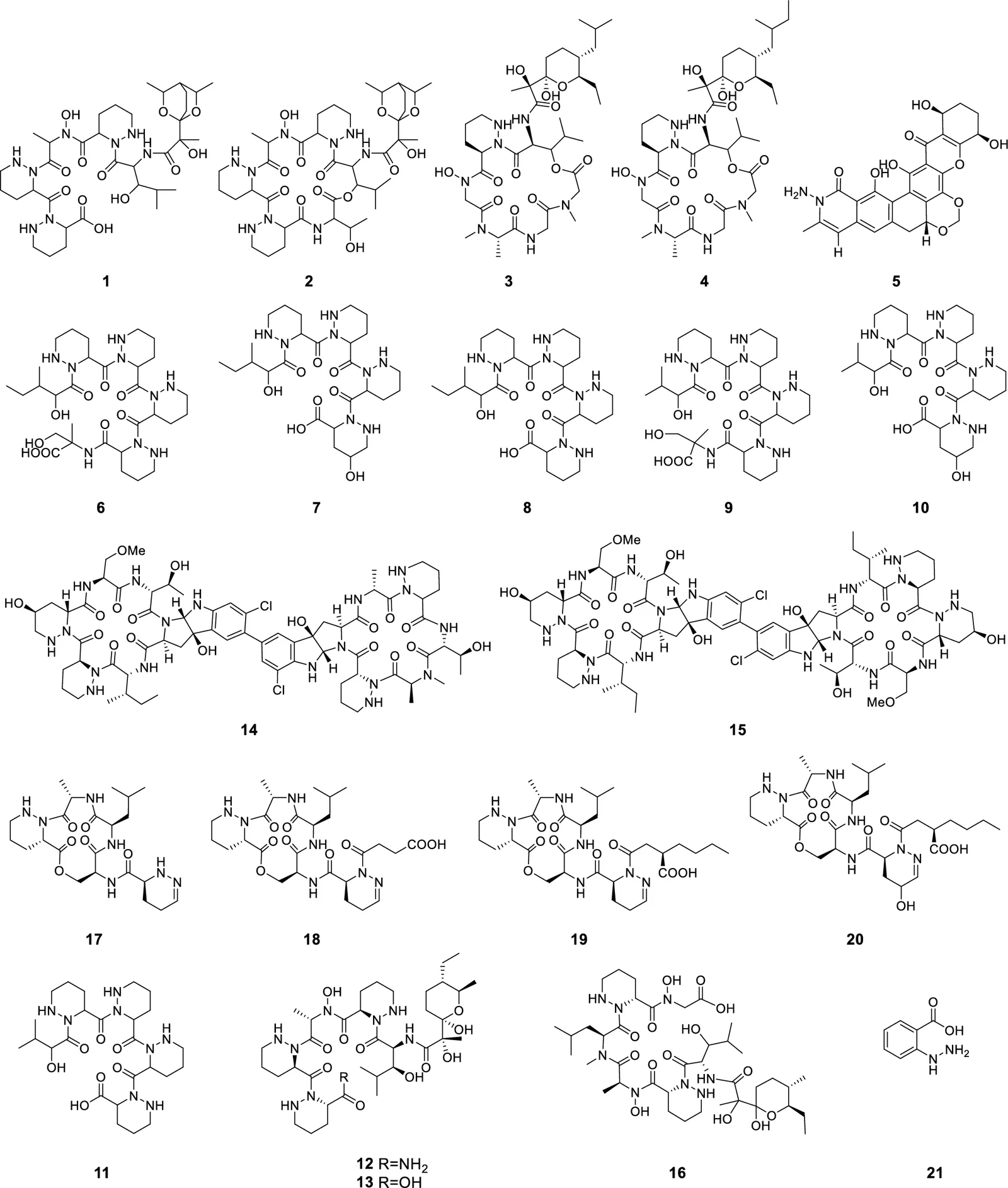
The chemical structures of compounds **1**–**21**

Acyclic amidine penipacids A–E were isolated from *Penicillium paneum* SD-44 in [35]. However, Capon's team questioned its structure, hypothesizing that the alternate stilbene structure was more in line with the spectral data. Interestingly, the researchers further hypothesized that this class of compounds formed artifact Schiff base adducts through the reaction of the potential hydrazine natural product *N-*aminoanthranilic acid (**21**) with solvents (acetone and methanol) and media-derived components (pyruvate and furfural). In vitro synthesis and comparison of spectral data verified that *N*-amino-phthalamic acid was the true natural product and corrected the structures of penipacids A–E (Fig. 1) [36].

PM130391 (**22**), PM130392 (**23**), and PM140293 (**24**) were isolated from *Streptomyces tuirus* in 2023. Notably, PM130392 was an isomeric mixture consisting of two peptides with different fatty acid chains. All three compounds were highly cytotoxic against lung carcinoma A549, colorectal carcinoma HT29, blast adenocarcinoma MDA-MB-231, and pancreas adenocarcinoma PSN1 cell lines, especially against the cell line of pancreas adenocarcinoma PSN1, among which, PM140293 showed the strongest toxic activity against this cell line and could be a candidate lead compound for the clinical treatment of pancreatic cancer [37].

Lunaemycin A (**25**) and lunaemycin B1 (**26**) were isolated from the moonmilk deposits of the cave of *Streptomyces lunaelactis* MM109^T^ production in 2023 [24, 38–40]. BGC 28a was predicted to produce piperazinic acid-containing non-ribosomal peptide metabolites. Consequently, two compounds were isolated. Among them, **26** is a dehydrogenated derivative of **25**. Meanwhile, the researchers proposed a hypothetical biosynthetic pathway for **25**. In addition, by constructing global natural products social molecular networking (GNPS) and HRMS/MS analysis, etc., compound **25** derivatives (lunaemycins B1–J3, **26**–**39**) were identified from two. *S*. *lunaelactis* (MM37 and MM109^T^). The **25**, **26**, and **30** showed growth inhibitory activity against some Gram-positive bacteria, especially **25** showed bactericidal and anti-resistant Gram-positive bacterial activity [24].

Lenziamides D1 (**40**) and B1 (**41**) were isolated from *Streptomyces* sp. S063 CGMCC 14582 in 2023. The gene cluster (*len*) for NRPS biosynthesis in *Streptomyces* sp. S063 was identified using NMR-based metabolomics and genome mining techniques and used as a basis for the isolation and purification of **40** and **41**. These two compounds exhibited moderate cytotoxicity against human cancer cell lines H1299, H1975, HEL and A549-taxol [41].

Polyoxyperuin B seco acid (**42**), depsidomycin D (**43**) and lenziamides A (**44**) and B (**45**) were isolated from *Streptomyces* sp. GB16, BYK1239, PC5 and SNJ018, respectively. Using primers targeting the piperazic-acid N–N bond-forming enzyme gene *ktzT*, 62 piperazinic-acid-producing strains were screened from a bacterial DNA library via PCR, followed by phylogenetic-tree analysis. LC–MS eliminated strains producing known piperazinic-acid derivatives, and subsequent ^15^NH_4_Cl-enriched fermentation combined with NMR spectroscopy enabled the isolation of new compounds and prediction of their biosynthetic pathways. Compound **44** showed strong growth inhibitory activity against tested human cancer cells, especially colon cancer cell lines, and also showed anti-tumor activity and antiproliferative activity against 5-FU-resistant colon cancer cell line in combination with 5-FU [8].

Caveamides A (**46**) and B (**47**) were molecules containing piperazine phosphate (Piz) isolated from *Streptomyces* sp. BE230 in 2024 by genome mining combined with ^15^N-NMR approach. Compound **47** can be converted to caveamides A by alkaline hydrolysis. Caveamides are the first peptides reported to contain a CHA residue, and the *N*-Me-CHA configuration was determined by stereoselective dibromination. Simultaneously, the prephenate decarboxylase (PDX) pathway [42] initiates the synthesis of CHA. Compound **47** exhibited greater cytotoxicity against HeLa, HepG2, LNCaP, and PC3 human cancer cell lines than caveamide A and in vitro antimicrobial activity against Gram-negative *E*. *coli*. and MRSA [43].

Diperamycin (**48**) was isolated in 2024 from *Streptomyces* sp. CS113, which belonged to the “CS” strain collection [44]. The “CS” strains are a collection of seventy-one *Streptomyces* strains isolated from cuticle of leaf-cutting ants [44, 45]. The research processed for the biosynthesis of diperamycin was also described in detail [44].

Depsidomycins B (**49**) and C (**50**) were two cyclopeptides isolated from *Streptomyces* sp. BYK1371 that contain D-leucine, D-threonine, D-valine, and S-piperazic structural units. Compounds **49** and **50** exhibited significant anti-metastatic activity against metastatic breast cancer cells (MDA-MB-231) [46].

Hydrazidomycin D (**51**) was a novel hydrazide compound isolated from a pair of actinomycetes. The new acylhydrazide compound was ultimately identified from a co-culture of *Streptomyces* sp. RKBH-B178 and heat-killed *Mycobacterium smegmatis*, following the addition of an autoclaved “inducer” microbial culture to the actinomycete fermentation medium. This compound exhibited cytotoxic activity exclusively against MCF7 breast cancer cells, with an IC_50_ value of 16 µg/mL [26].

**Fig. 2:**
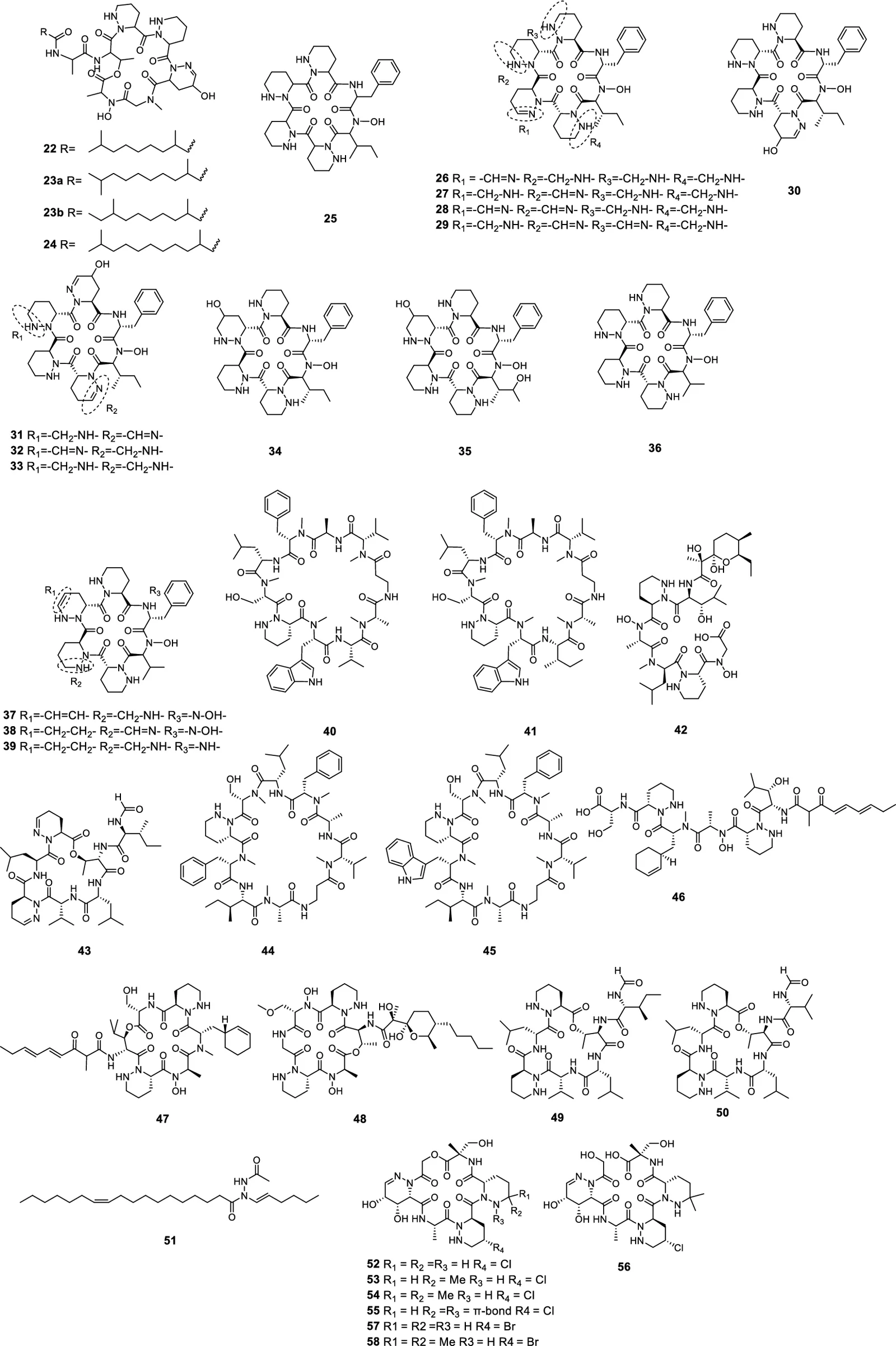
The chemical structures of compounds **22**–**58**

Svetamycins A–G (**52**–**58**) were halogenated peptides isolated from *Streptomyces* sp. Among them, svetamycin E is a linear analog of svetamycin C, while the others are cyclic peptides. The svetamycins feature a hydroxyl acetic acid and five amino acids among which are the uncommon 4,5dihydroxy-2,3,4,5-tetrahydropyridazine-3-carboxylic acid and a *γ*-halogenated piperazic acid. represents the first instance of a piperazyl scaffold adopting a pseudoequatorial conformation of the *α*-carboxamide group within a peptide framework. Among these, compounds **52**, **54**, and **57**–**58** exhibited activity against both *M. smegmatis* and MRSA. The **58** showed the strongest inhibitory activity against *M. smegmatis* (IC_80_ = 2 μg/mL), while **54** and **58** exhibited the best antibacterial effects against MRSA. However, compound 5 showed virtually no activity (Fig. 2) [47].

#### Hydrazines

Peniproline A (**59**) was isolated from an endophytic fungus *Penicillium decumbens* CP-4 associated with medicinal plants in 2017. Peniproline A was structurally the first natural compound to possess a 1-phenylamino-2-pyrrolidone unit, and its N–N bond was due to the coupling of a phenylamine unit with 2-pyrrolidone. However, Peniproline A did not show strong cytotoxicities against Hela cells lines (IC_50_ 15.5 μM) and Bel-7402 (IC_50_ 8.1 μM) [48].

FSTR (**60**) and difluostatin B (**61**) were new compounds produced by introducing the *fls* gene cluster into the heterologously expressed strain *Streptomyces albus* J1074 in 2018. Dimer formation of difluostatin B was via spontaneous deacylation of acyl-containing FSTs to generate a p-quinone methide (p-QM) intermediate, followed by dimer formation via C–N bond coupling with another not-yet-isolated FST analog nucleophilic reagent, while the epoxide C-2 site was mediated by C-6′ OH to participate in the ring-opening reaction without enzyme catalysis [49].

**Fig. 3:**
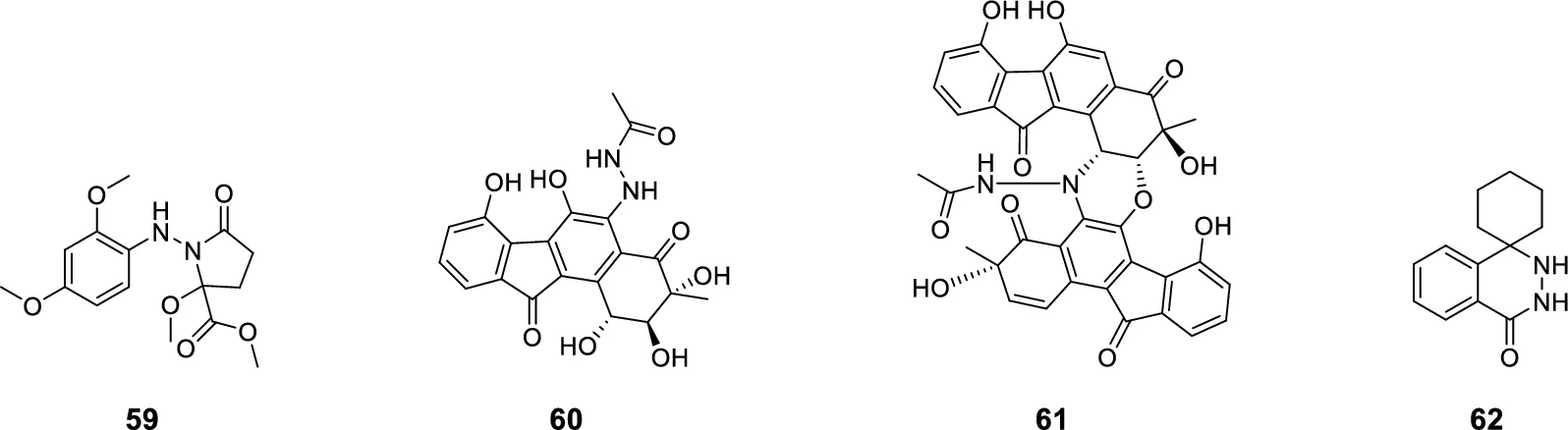
The chemical structures of compounds **59**–**62**

Amycophthalazinone A (**62**) was isolated from *Amycolatopsis* sp. YIM 130642 associated with lichens and was also the first natural phthalazinone derivative to be discovered. Using the broth microdilution method, amycophthalazinone A had strong inhibitory activity against *Candida albicans*, *Salmonella typhi* and *Staphylococcus aureus* (Fig. 3) [50].

#### Hydrazones

Farylhydrazone C (**63**) was isolated from *Penicillium* sp. HDN14-431 that was discovered in 2016 from the soil of the Antarctic mesolittoral zone. However, the compound was not cytotoxic to the cancer cell lines tested and showed antimicrobial activity only against *Proteusbacillus vulgaris* tested [51].

Amino group carrier protein (AmCP) could bind to the α-amino group of the substrate and participated in the biosynthesis of DADH or its derivatives. Therefore, the new natural product s56-p1 (**64**) was identified by PCR screening and heterologous expression of the *AmCP* gene in *Streptomyces lividans* TK23 in 2017. The researchers proposed the putative biosynthetic pathway [52]. Subsequently, Nishiyama's team proposed a machinery for hydrazine formation in s56-p1 biosynthesis [53].

Prolinimines A–D (**65–68**) were isolated from the rice medium of a fish gastrointestinal tractderived fungus *Trichoderma* sp. CMB-F563 in 2018, and represented the natural product known to possess the *L*-pro and hydrazinyl Schiff bases isolated from microorganisms. Compound **65** was converted to compounds **67** and **68** by acid-mediated conversion. Marfey’s analysis [54] showed that Compound **66** had the *L*-configuration for pro and the *E*-configuration for imine as determined by diagnostic ROESY. In addition, compounds **65** and **66** were biomimetic synthesized with L-pro as the origin [55].

Murayaquinone C (**69**) was isolated from the insect-derived bacterium *Streptomyces* sp. NA4286 in 2019. However, the compound showed no cytotoxicity against the tested human cancer cell lines, probably due to the bulkier size of the C-12 substituent which affected the activity [56].

Penoxahydrazones A–C (**70–72**) were isolated from the fungus *Penicillium oxalicum* that was discovered in 2021 from the deep-sea cold seep. Compound **70** was the first known phenylhydrazone-bearing steroid natural product and the absolute configuration determined by X-ray crystal diffraction. It was worth noting that compounds **71** and **72** were very rare phenylhydrazone tautomers. Among them, compound **70** showed significant inhibitory activity against the tested microalgae, moderate inhibitory activity against *Vibrio harveyi*, *Vibrio parahaemolyticus* as well as low toxicity against marine zooplankton *Artemia salina* [57].

Tasikamides A–C (**73–75**) were isolated from *Streptomyces tasikensis* P46 and mainly composed of a cyclic peptide, an alkyl 5-hydroxylanthranilate (AHA) moiety and a hydrazone group. Of these, BGC22 (*tsk BGC*) and *aha* BGC were responsible for the biosynthesis of the cyclic peptide portion and the AHA, respectively. Notably, the N=N bond formation in diazo-AHA was formed by Aha1 and Aha2 providing nitrite as the nitrogen source through the ANS pathway followed by Aha11 diazotizing the AHA. Ultimately, diazo-AHA and the cyclic peptide portion were linked by Japp–Klingemann reaction in the form of C=N Bond thereby forming tasikamides A–C [58].

Penzonemycins A–B (**76–77**) were isolated from *Streptomyces* sp. SCSIO 40020 in 2022. The absolute configuration of compound **76** was determined by X-ray diffraction experiments. The diazo (N=N) and final hydrazone moiety (− N−N=C −) of this compound were formed similarly to those of tasikamides. The compound **76** showed weak cytotoxicity against human cancer cell lines SF-268, A549 and HepG-2, in addition to the absence of antifungal activities, antibacterial activities and α-glucosidase inhibition activity [59].

**Fig. 4:**
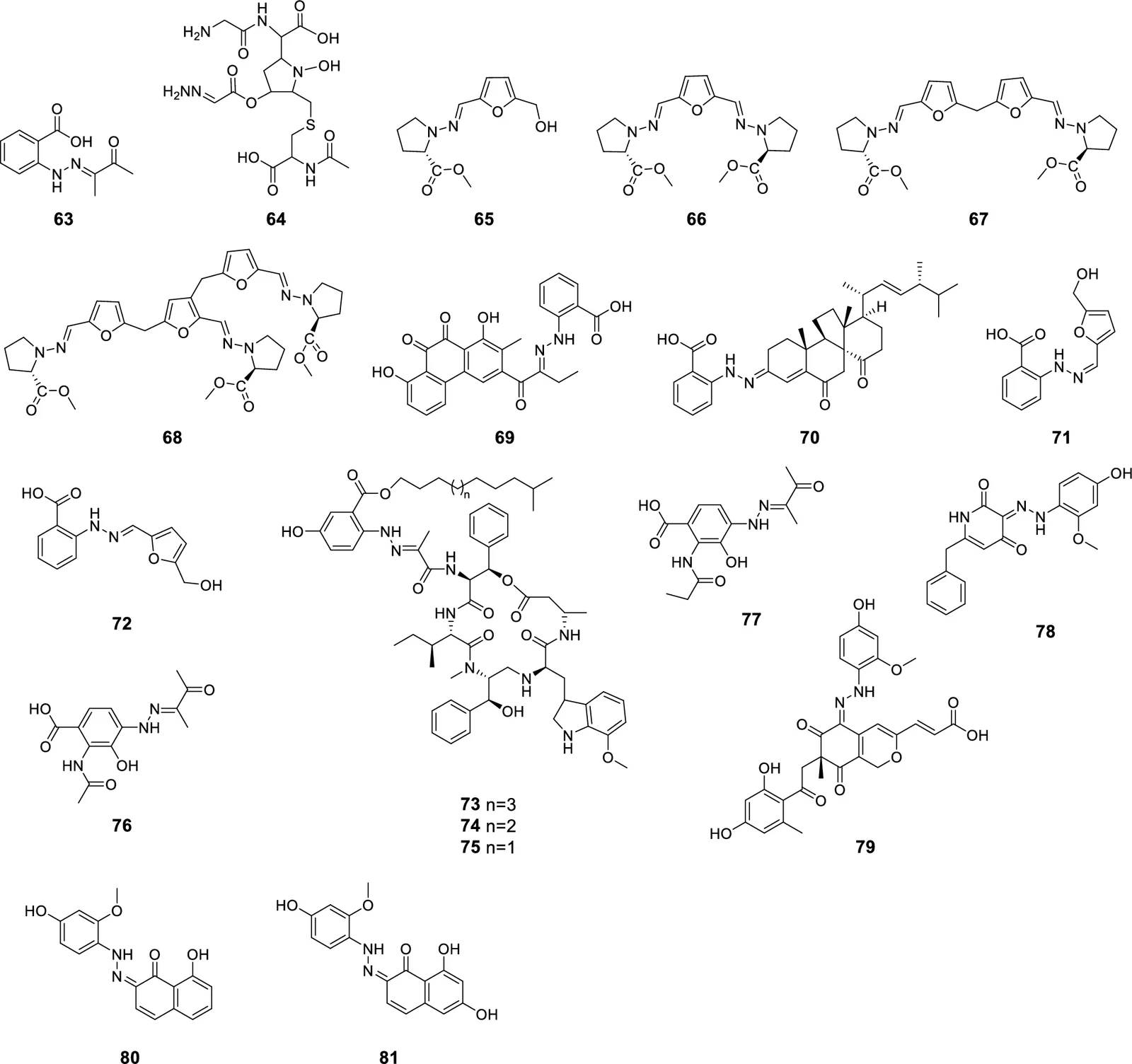
The chemical structure of compounds **63**–**81**

Talarohydrazones A–D (**78–81**) were phenylhydrazone alkaloids isolated from fungus *Talaromyces amestolkiae* HDN21-0307 in 2024 utilizing the one strain-many compounds (OSMAC) approach in combination with GNPS MN, NAP and MS2LDA. Notably, compound **78** has the first naturally occurring phenylhydrazone-pyridinedione hybrid skeleton and is known to possess a naturally rare 2,4-pyridinedione moiety. The **79** represented the first known azadophilone−phenylhydrazone skeleton natural product. Compound **78** showed cytotoxicity against NCI-H446 and antimicrobial activity against *Staphylococcus aureus* ATCC 29213. The other compounds did not possess significant cytotoxicity and antimicrobial activity (Fig. 4) [22].

#### Azo and azoxy compounds

Poroikov's team summarized in 2017 about azo compounds and their derivatives among microorganisms. Structures and biological activities of the compounds, etc. could be found in this literature [60].

KA57A (**82**) was isolated and discovered by constructing a triple mutant KA57 (Δ*srrB*·Δ*lkmE*·*lkcF*-*KR1*^Y343F^). Notably, this strategy activates the silent biosynthetic clusters in *Streptomyces rochei* leading to the discovery of a new natural product. The C-2 stereochemical configuration of this compound was deduced to be the *R*-configuration. In addition, the researchers resolved the origin of the carbon and nitrogen of the compound and proposed a putative biosynthetic pathway [61].

Azoxymycins A–C (**83–85**) were isolated from *Streptomyces chattanoogensis* L10 in 2015. Due to *trans* or *cis* isomeric transitions of the double bond and migration of oxygen atoms between the two nitrogen atoms in the azoxy bond, especially under UV or heat conditions, compounds **83** and **85** consisted of two exchanging isomers and azoxymycin B consisted of a mixture of four interconjugated isomers. In the biosynthesis, compounds **83** and **85** were formed by the AzoC-catalyzed azoxy group linkage of two identical or different precursors [62].

Azodyrecins A–C (**86–88**) were isolated from *Streptomyces* sp. strain P8-A2 and identified. The *cis*-alkenyl unit of azodyrecins A–C was relatively easy to isomerize, forming the *trans*-isomers. Furthermore, L-alanine and alkylamines could be used as a source of initiation for the biosynthesis of compounds **86–88**. Moreover, in cytotoxicity assays, compound **86** was resistant to brine shrimp *Artemia salina* and compound **87** was cytotoxic to human leukemia cell line HL-60 [63].

Azodyrecins D–G (**89**–**92**) were isolated from two actinomycete strains *(Streptomyces* sp. RM72 and *Streptomyces* sp. A1C6) using a screening approach guided by specific chemical probes. The core of this method involves first identifying strains through genomic screening, then using N2H4—produced by the hydrolysis of azo compounds—as an indicator signal to identify the compounds. Unfortunately, these compounds exhibited virtually no significant cytotoxic activity [64].

*O*-Alkylazoxymycins A–F (**93–98**) were isolated and characterized from *Streptomyces* sp. Py50 in 2023. The biosynthesis of **94** and **95** was based on the use of L-lysine and anthranilic acid as precursors, in which L-lysine underwent decarboxylation, hydroxylation by monooxygenase, and condensation to produce one intermediate, and anthranilic acid produced another intermediate by condensation with malonyl-CoA. The two intermediates were condensed to form an azoxy bond via *N*-oxygenase to form **94** and **95**. In addition, the **93**, **97** and **98** were active against *Epidermophyton floccosum* [65].

**Fig. 5:**
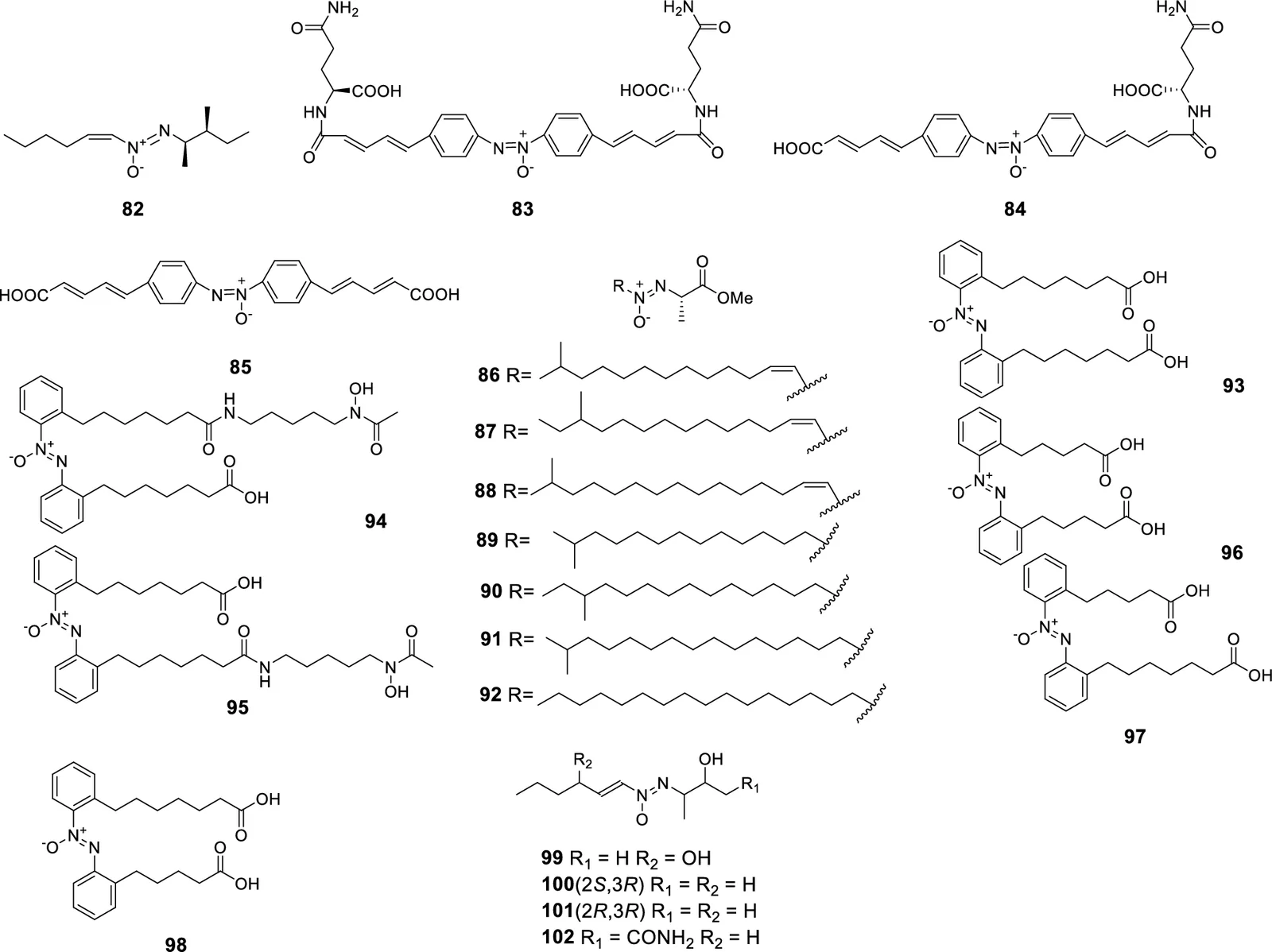
The chemical structures of compounds **82**–**102**

Maniwamycins C–F (**99**–**102**) were isolated from the culture broth of *Streptomyces* TOHO-M025. Notably, maniwamycins can dose-dependently inhibit the synthesis of the purple pigment (*Chromobacterium violaceum* CV026) regulated by quorum sensing. The **98** and **99** are more potent than **97** and **100**. In addition, maniwamycins exhibited antimicrobial activity against *C. violaceum* CV026, *Staphylococcus aureus* TCC25923, *Pseudomonas aeruginosa* ATCC27853, *Escherichia coli* ATCC25922, *Candida albicans* JCM1542, and *Aspergillus flavus* JCM1738 (Fig. 5) [66].

#### Nitrosohydroxylamines

Gramibactin (**103**) was the first *N*-Nitrosohydroxylamines-containing novel siderophore isolated from *Paraburkholderia graminis* by genome mining in 2018. The building blocks of the *D*-configuration of this compound could be determined by a modified Marfey method. Surprisingly, plants could utilize the iron dissolved by the iron carrier Gramibactin, which promoted chlorophyll synthesis in plants [67].

By genome mining of grbD in gramibactin, which was presumably involved in the formation of N–N bonds, gramibactin B (**104**) and megapolibactins A–F (**105**–**110**) were found in the plant mutualists *Paraburkholderia. caledonica* and *P. megapolitana*, respectively. They were categorized into two groups and one subgroup. Among them, the subgroup consisted of two cyclic megapolibactins, including megapolibactin A_cyc_ (**111**) and megapolibactin B_cyc_ (**112**). In addition, plantaribactin (**113**) was also isolated from the plant pathogenic bacterium *Burkholderia. plantarii*. A homologue of plantaribactin, gladiobactin (**114**), was also identified in *B. gladioli* by gene cluster similarity [67].

Trinickiabactin (**115**) was isolated from *Trinickia caryophylli* DSM 50341 in 2020 and is a linear hexapeptide structure. It was mainly biosynthesized by *trbHIJ* encoding non-ribosomal peptide synthetase (NRPS), which was highly similar to *grb* BGC of gramibactin. The **115** exhibited strong antimicrobial activity against *Yersinia pseudotuberculosis* as well as low and moderate activity against *Pseudomonas aeruginosa* PA14 and *Burkholderia thailandensis* DSM 13276, respectively [68].

**Fig. 6:**
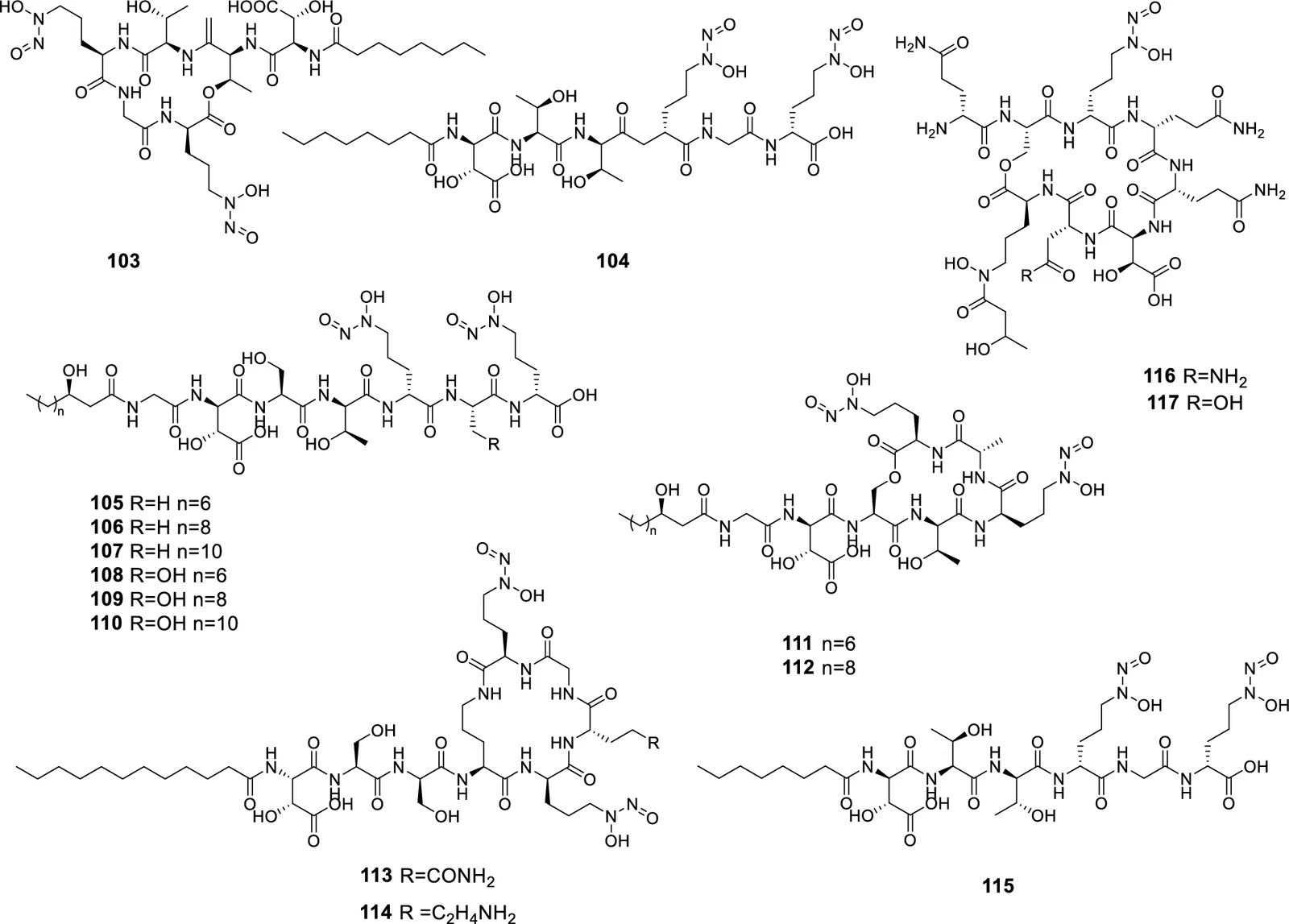
The chemical structures of compounds **103**–**115**

The iron carriers tistrellabactins A and B (**116**–**117**) were discovered in marine-derived *Tistrella mobilis* KA081020-065 by gene cluster mining and isolated by purification under Fe-limited conditions in 2023. The *C*-diazeniumdiolate of apo-tistrellabactin was converted to oxime isomers and released NO upon photolysis, while two other ligands (hydroxamate and β-OH-Asp) retained the ability to coordinate Fe(III). However, two ligands (β-OH-Asp and *C*-diazeniumdiolate) of the Fe(III)-tistrellabactin A lost their ability to coordinate Fe(III) under prolonged photolysis and released free iron, which can then participate in marine iron cycling reactions and other processes (Fig. 6) [69].

#### Nitrogen-containing heterocycles

WS9326H (**118**) was isolated from mudflat-derived strain *Streptomyces* sp. SNM55, and was the first discovery of a cyclic heptapeptide with a specific pyrazolone ring. The pyrazolone ring is connected to a *D*-arabinitol through an amide bond, which was very rare in nature. Furthermore, the stereochemical configuration of WS9326H was determined by Marfey’s analysis [70], ROESY, and DP4 calculations, etc. Although WS9326H was not cytotoxic to the five cancer cells tested, it exhibited antiangiogenic activity [71].

By constructing a mutant of the *flsP* gene encoding a FAD-binding oxidoreductase in *Micromonospora rosaria* SCSIO N160, the difluostatin I (**119**) is the first discovery of a natural heterodimer of 3′,4′-*seco*-fluostatin. The biosynthetic mechanism of the dimerization was similar to difluostatin B, which also involved the generation of a p-QM-like intermediate by a nonenzymatic deacylation reaction followed by a C–N bond coupling with another pyrazolofluostatin homologue [72].

Delivery of deuterated fatty acids (FAs) d_*11*_-C_*6*_ to the cyanobacterial *Nodularia* sp. LEGE 06071 in the absence of the β-oxidation pathway allowed the incorporation of d_*11*_-C_*6*_ into metabolites for fatty acid chain elongation, leading to the discovery of nocuolactylates A–E (**120**–**124**), a new class of 1,2,3-oxadiazine-lactylate hybrids with a presumed absolute configuration of 4*R*,18*S*. Compounds **120** and **121** exhibited cytotoxicity against the cell lines tested, and in addition the nocuolactylate A exhibited antimicrobial activity against *Bacillus subtilis* [73].

Three new cytochalasan derivatives chaetoglobosins B1-B3 (**125**–**127**), were isolated from the crude broth extract of *Chaetomium madrasense* 375 after chemical engineering via reaction with hydrazine monohydrate. The **125** exhibited selected antiproliferative activity on non-small-cell lung carcinoma A549 [74].

Pyrazolofluostatins A–C (**128**–**130**) were isolated from the South China Sea-derived *Micromonospora rosaria* SCSIO N160, and possessd a benzo[*cd*]indeno[2,1-*f*]indazol skeleton with a pyrazole-fused 6/5/6/6/5 pentacyclic ring system, representing a completely new carbon skeleton structure. Among them, the **128** exhibited moderate antioxidation activity (EC_50_ 48.6 μM) [75].

**Fig. 7:**
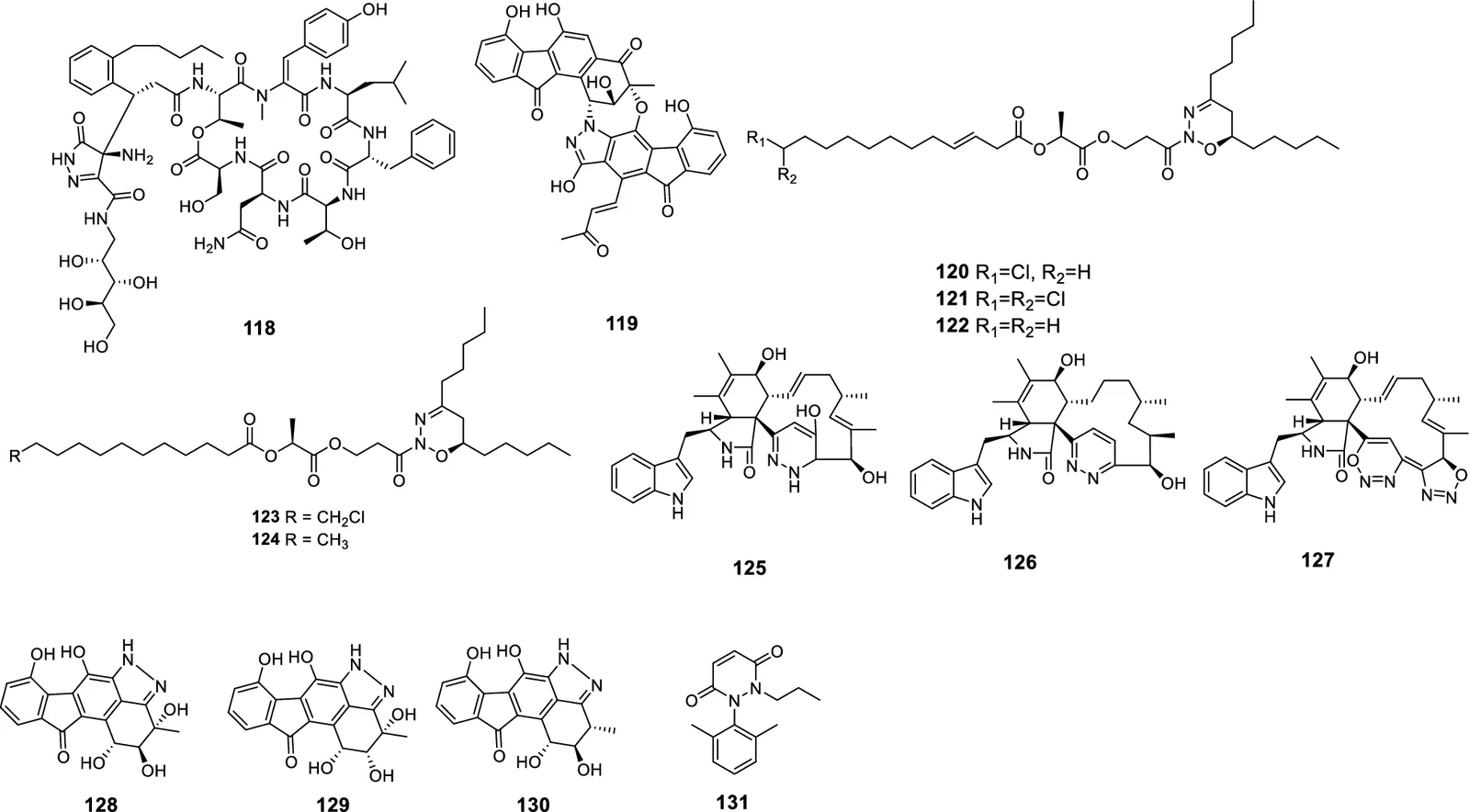
The chemical structures of compounds **118**–**131**

A new phenyl pyridazine derivative (**131**) was isolated from the fungus *Aspergillus* sp. AV-2 sourced from mangrove forests. Unfortunately, the compound did not exhibit significant cytotoxic activity (Fig. 7) [76].

Among the 131 compounds, Hydrazides are overwhelmingly dominant (44.27%), representing the most frequently reported class of N–N bond-containing natural products during this period. Hydrazones (14.50%) and Azo and Azoxy Compounds (16.03%) rank second and third, respectively. Nitrosohydroxylamines account for 11.45%, while N–N-containing heterocycles (e.g., pyrazoles, triazoles) and hydrazines are less abundant, representing 10.69 and 3.05%, respectively.

**Fig. 8:**
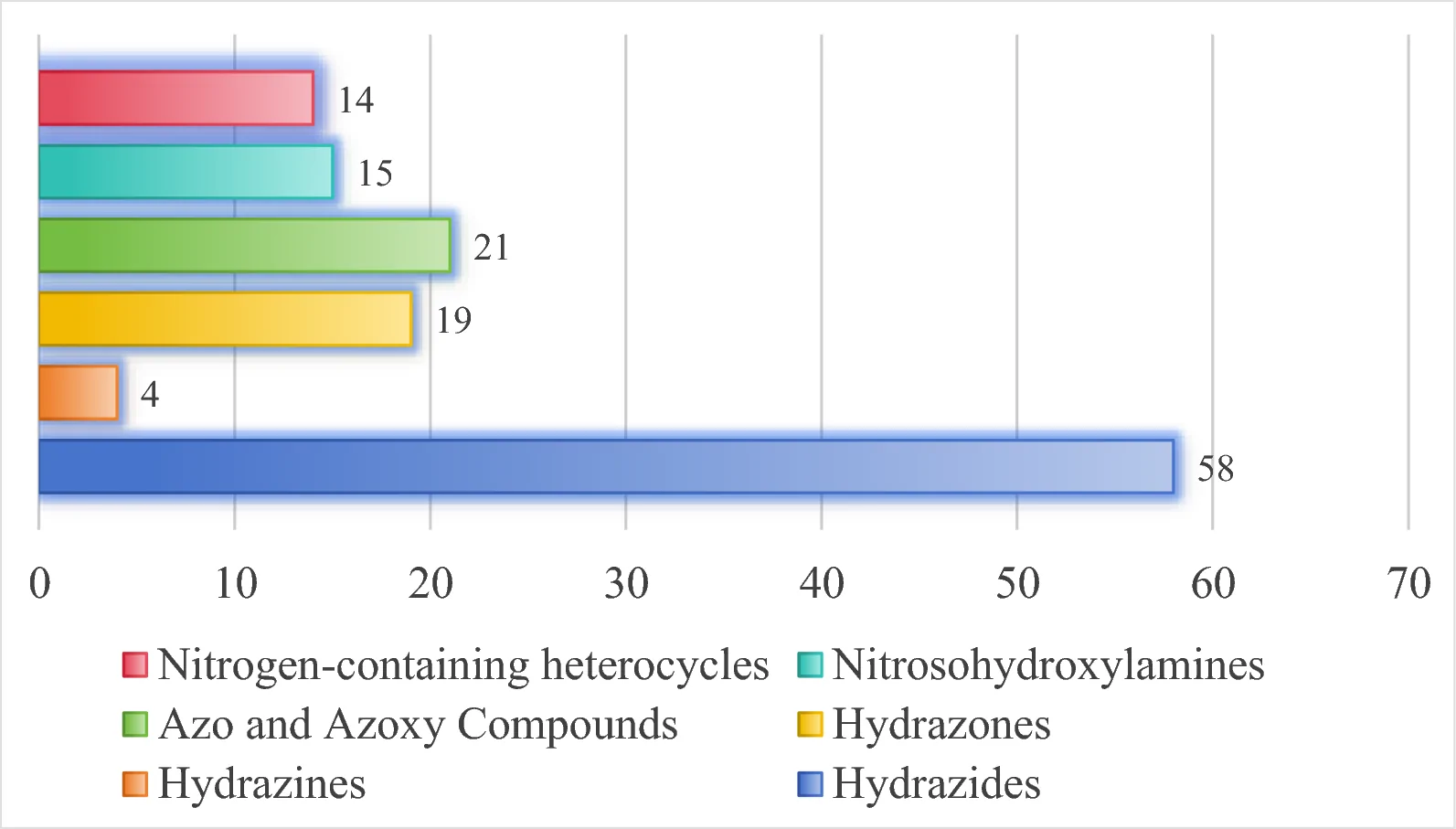
Statistical distribution of 131 N–N bond natural products by core functional groups, 2014–2024

Piperazic acid in Hydrazides are the non-proteinogenic amino acid containing an N–N single bond, usually embedded in nonribosomal peptides or polyketide–peptide hybrids. Its high occurrence suggests that the enzymatic machinery for piperazic acid formation (e.g., *N*-amination and oxidative cyclization) is broadly proficient in microbial secondary metabolism, particularly in actinomycetes and fungi (Fig. 8).

## Bioactivity and pharmacological analysis

### Pharmacology of verucopeptin

Multidrug resistance (MDR) is the development of an acquired resistance in cancer cells and microorganisms by chemotherapeutic drugs with different mechanisms of action and chemical structures [77]. MDR mainly pumps different types of amphipathic drugs out of the cell through overexpressed the ATP-binding cassette (ABC) transporters, which reduces the concentration of the drugs in the cell, thus affecting the therapeutic efficacy. Especially among cancer treatments, MDR may occur with multiple chemotherapies, and even cross-resistance to drug molecules of unrelated structures may occur, adversely affecting cancer treatment [77]. As a result, researchers have focused specifically on a number of ways to inhibit MDR [78, 79].

Recently, Deng and his collaborators performed a cytotoxicity screen on the vincristine-induced MDR gastric cancer cell line SGC7901/VCR for verucopeptin (VE) derived from *Actinomadura* sp. XM-4-3 from the microorganism's internal natural product library. VE exhibites a broad spectrum of anti-proliferative activity and tissue specificity in vivo and in vitro. By using cytological profiling, VE is found to induce apoptosis, and further KEGG analysis after treatment of VE-resistant U2OS cells revealed that VE interfered with the lysosomal pathway, which may be related to the cytotoxic effects. The researchers further designed a photoactive chemical probe VE-P. By labeling VE-P and lysosomes, it is found that VE-P colocalized with lysosomes, which further suggested that there may be a direct correlation between VE and lysosomes [80].

The researchers identified the highest percentage of precipitates formed by VE-P binding to the ATP6V1G subunit through a chemical proteomic strategy of click chemistry [81] and gene ontology (GO) analysis of differential lysosomal proteins. To verify whether VE-P binded specifically to the ATP6V1G subunit, the researchers demonstrated the validation using a competitive binding assay and immunoprecipitation assays with an anti-ATP6V1G1 antibody and inhibition of enzyme activity. Meanwhile, the construction of amino acid mutants of the ATP6V1G subunit not only identified the key amino acid residues (L99S/C104Y), but also demonstrated VE inhibites v-ATPase activity by specifically targeting the ATP6V1G1 subunit [80].

Earlier studies have demonstrated that mTORC1 could be recruited to the lysosomal surface through activation of Rag GTPase, which served as a scaffolding platform for mTORC1 activation [82–87]. In HL60 cells, VE significantly inhibites the phosphorylation of mTORC1 downstream substrates in both the 10–200 nM concentration and 50–500 nM concentration ranges. Meanwhile, further immunoprecipitation assay and cell-free experiment demonstrated that VE dissociated the binding of mTORC1 from Rag GTPases on the surface of lysosomal surface [80, 86]. Subsequently, by constructing A375 and U2OS RAG^active^ cells stably expressing Rag A/B^GTP^ and RagC/D^GDP^, as well as ATP6V1G1^L99S^ and ATP6V1G1^C104Y^ mutations, it is found to be able to efficiently resist VE-induced downregulation of mTORC1 signaling, suggesting that VE targeted ATP6V1G1, thereby down-regulating mTORC1 that utilized a Rag-dependent lysosomal machinery [80].

In SGC7901/VCR and K562R cells, the most potent antiproliferative capacity of VE compared with other v-ATPase inhibitors and the most effective way to inhibit both mTORC1 signaling and v-ATPase activity may be achieved by targeting the V1G subunit of v-ATPase [80].

Finally, the researchers established a mouse model of xenografting with SGC7901/VCR cells. By giving the appropriate dose, frequency and time, it is found that VE significantly inhibites tumor growth and mTORC1 signaling. It is able to induce cell death, but no significant side effects were seen, suggesting that VE also has an anti-MDR tumor effect in vivo. Therefore, VE can be used as a lead compound for the treatment of MDR cancers in the future, and further studies such as clinical trials can be conducted [80].

Beyond verucopeptin, numerous N–N bond-containing compounds exhibit promising bioactivities. Piperazic acid-containing cyclic peptides display anticancer activity, including petrichorins, lydiamycins F–H, PM140293 and caveamide B. Antibacterial activity has been reported for meliponamycins A–B and albofungin A. Siderophore-type compounds such as gramibactin, while other activities include anti-microalgal (penoxahydrazone A), antiangiogenic (WS9326H) effects. It is worth noting that, for most of these compounds, only data on cytotoxicity or antibacterial activity have been reported to date, and their molecular targets and detailed mechanisms of action remain unclear. Therefore, further research using techniques such as molecular docking is still needed to elucidate the molecular mechanisms and advance drug development.

### Structure–activity relationship (SAR) of N–N bond-containing natural products

By integrating existing activity data on compounds containing N–N bonds, this paper summarizes relevant SAR rules for metabolites, identifying N–N-adjacent substituents, the core ring skeleton, and the type of N–N bond as the three key factors influencing their anticancer and antibacterial bioactivities.

First, effect of N–N adjacent substituents. Caveamides A and B share the same peptide backbone. Caveamide A adopts an open-chain β-ketoamide form, while caveamide B undergoes spontaneous intramolecular cyclization. The newly-formed ring creates an extra polar hydrogen-bonding site adjacent to the piperazic acid N–N backbone, which may promote binding to intracellular target proteins and thereby markedly increase cytotoxicity relative to caveamide A [43]. For cyclic svetamycins, svetamycin G is more potent than svetamycin F and svetamycin C is more active than svetamycin A. These findings suggest that *δ*-methylation of piperazic acid enhances potency. In addition, svetamycin G is more active than svetamycin C, which proves that bromine substitution for chlorine improves antibacterial activity. Svetamycin E, with its open ring, loses its activity, suggesting that a macrocyclization reaction is necessary for it to exhibit antibiotic activity.

Secondly, core skeleton difference. Cyclic peptides containing piperazine acid typically exhibit significant antitumor activity, whereas simple heterocyclic compounds primarily exhibit weak antibacterial activity. This is mainly due to the rigid conformation of cyclic peptides, providing a large number of hydrogen bond donors and acceptors and enabling them to form strong interactions with tumor targets. At the same time, cyclic peptides are not easily degraded by proteases in vivo, making the configuration more stable. Verucopeptin, which is a cyclopeptide containing multiple piperazine acid units, can precisely target the ATP6V1G subunit, potently inhibiting v-ATPase activity and reversing MDR [80].

Thirdly, N–N bond types. In addition to the skeletal conformation, the electronic properties of different N–N bonds may result in markedly different biological activities [88]. The saturated N–N single bond in piperazine acid exhibits moderate electron-donating properties and, through synergistic interaction with surrounding peptide residues, provides a targeted and specific binding pocket for tumor-associated proteins. In contrast, the azoxy (–N=N–O–) group tends to interact more with bacteria. Natural products of the azoxy class, such as *O*-Alkylazoxymycins, primarily exhibits antibacterial activity. Whereas cyclopeptides, as exemplified by verucopeptin, exhibit broad-spectrum antitumor selectivity.

## Related enzymes catalyzing the formation of N–N bonds

### Formation of N–N Bonds from nitrite substrates

Feeding experiments with azamerone revealed that nitrite can be involved in the formation of N–N bonds [89], but the exact mechanism remains unclear [15]. In recent years, biosynthetic studies of cremeomycin have led to the discovery of the source of nitrite and the processes involved in its synthesis. Both Waldman et al. and Sugai et al. [90, 91] have proposed 3-amino-4-hydroxybenzoic acid (3,4-AHBA) as a biosynthetic intermediate. Waldman et al. [90] primarily established the sequence of reactions in cremeomycin synthesis. Searching for cremeomycin (*cre*) biosynthesis gene clusters by using a chemically guided genome mining approach. Sugai et al. [91] determined that CreE and CreD are involved in the late synthesis of cremeomycin by knockout experiments. CreE and CreD were then analyzed in vitro to screen for amino acids that act as the nitrogen donor. The result showed that *L*-aspartic acid, as a substrate, was oxidized by CreE using NADPH as a reducing agent and detected to produce nitrosuccinic acid, and CreD catalyzed the nitrososuccinic acid to produce nitrous acid and fumaric acid. In Waldman et al. [92] demonstrated through in vivo heterologous expression, targeted mutagenesis, and in vitro experiments that 3,2,4-AHMBA and nitrite generated cremeomycin through the diazoxide-forming enzyme CreM, which depletes ATP in this process. This *L*-aspartate-derived nitrite-generating pathway mediated by CreE and CreD was formally named the ANS pathway [93].

The FzmL/FzmM homologs from fosfazinomycin BGC and Alp2F/Alp2G homologs from kinamycin BGC function via the classical ANS pathway. Unlike the discrete CreE/CreD system, the Fzm and Alp/Kin system require glutamate as a hydrazine carrier to transport acetylhydrazine derived from nitrite, which is then converted into fosfazinomycin and kinamycin through distinct biosynthetic pathways [94, 95]. In contrast to the independent Fzm system, the biosynthesis of kinamycin relies on a complete set of auxiliary Alp enzymes (Alp1I-Alp1N, Alp1S, Alp1V, Alp1T, Alp1U, Alp2D) to form the core diazobenzo[a]fluorene aromatic polycyclic skeleton. Subsequently, the conserved KinLKJNM protein generates the glutamylhydrazide, which is ultimately conjugated to the polycyclic skeleton to form kanamycin [94–96].

Tri16/Tri21 serve as CreD/CreE homologs for triacsin biosynthesis, producing nitrite that assembles into the third nitrogen of the *N*-hydroxytriazene moiety [97]. For alazopeptin, AzpE/AzpD are ANS pathway homologs [98–101]. The difference is a unique diazotase AzpL actes as a diazotrophic synthase to promote diazotrophic reaction between nitrite and 5-oxoLys, resulting in the production of diazo group [102].

In the biosynthesis of *L*-alanosine, both Balskus [103] and Ryan's teams [104] identified biosynthetic gene clusters for L-alanosine, named *ala* and *aln* gene cluster, respectively. AlaJ/AlaI (AlnM/AlnN) are ANS homologs. Differentiating from other ANS-dependent metabolites, L-aspartic acid-produced NO_2_^−^ exclusively contributes to the distal N-nitroso nitrogen atom of L-alanosine’s diazeniumdiolate core [103].

In some natural products containing diazeniumdiolate moiety, such as fragin, valdiazen [105, 106]. Current chromatographic evidence suggestes that the distal N atom of the diazeniumdiolate moiety is derived from nitrite [105]. This system lacks CreE/CreD homologs entirely. Instead, HamC is predicted to provide the distal oxidized nitrogen atom of the diazeniumdiolate moiety [106]. However, the involvement of nitrite and the enzyme that catalyzes the formation of the diazeniumdiolate requires further investigation [105, 106].

### Formation of N–N bonds catalyzed by hydrazine synthase

Hydrazine synthetases generally consist of a *C*-terminal methionyl-tRNA synthetase (MetRS-like domains) and an *N*-terminal zinc-binding cupin domain [12]. Direct N−N bond formation is disfavored by nitrogen’s high electronegativity. Thus, one nitrogen atom needs enhanced electrophilicity to accept nucleophilic attack for N−N linkage construction [13]. The reaction mechanism is mainly carried out in two parts. The first step is played by MetRS-like domains, which determines the specificity of the amino acid. Firstly, the amino acids are activated by analogs of the typical amino acyl-tRNA synthetases by consuming ATP, and the activated amino acids are coupled with the *N*ω-hydroxylamine substrate to generate the *O*-acylhydroxylamine intermediate, and the second part is played by the zinc-binding cupin domains, which converts the generated intermediate into hydrazine molecule, forming a N–N bond [12].

*N*-hydroxylating monooxygenases (NMOs) determine the specificity of hydroxylamine substrates, containing not only common amino acids, but also cadaverine and putrescine [107]. Representative enzymes include KtzL/Por9/VlmH for ornithine, lysine and isobutylamine, respectively [108] and Spb38/Tri26/ForK/PyrM for lysine hydroxylation. A FAD-dependent ornithine *N*-monooxygenase MbaC catalyzes the formation of hydroxyornithine from ornithine and incorporates it into NRPS for the synthesis of the malleobactin B intermediate, which may subsequently be non-enzymatically autoxidized or dimerized to form malleobactins [109].

Besides nitrite synthesis, NMO-generated hydroxylamines can condense with carboxyl groups to form N–N-containing compounds [107]. Zhao and Toshiyuki et al. demonstrated that some hydrazine synthases can react with specific hydroxylamine substrates to generate a range of mixed hydrazine products [6, 12].

PyrN and its homologs (Spb40/Tri28) use diverse amino-acid substrates (L-glutamate, L-glycine, L-serine, L-alanine, and L-tyrosine) to form N–N bonds with *N*^6^-OH L-lysine [6]. Furthermore, hydrazine synthase have a broad tolerance to hydroxylamine substrates, For example, hydrazine Apy9 reactes with *N*^6^-OH L-lysine and *N*^4^-OH L-diaminobutyric acid (l-DABA), but DABA-*N*^4^-hydroxylase Apy11 acts as a gatekeeper and specifically hydroxylates *L*-DABA, so that Apy9 specifically recognizes *N*^4^-OH L-DABA provided by Apy11 [108]. Thus, NMOs and hydrazine-synthase MetRS-like domains govern amine precursor selection for N–N bond biosynthesis [12].

After the formation of hydrazine intermediates, site-selective C–N bond cleavage on both sides of the N–N bond by flavin-dependent oxidases is also required, and the broken portion can be reutilized in hydrazine synthesis as a nitrogen carrier after passing through the primary metabolic cycle [12].

The gene cluster (*spb*) for the biosynthesis of the hydrazone natural product s56-p1 is the first to be identified [53]. Nishiyama et al. demonstrated its biosynthetic pathway through gene knockout, heterologous expression, and in vitro recombinant enzyme experiments [53]. *N*-hydroxylase Spb38 first hydroxylates L-lysine to form *N*^6^-OH L-lysine, which then formes a hydrazine intermediate conjugated with glycine in the presence of hydrazine synthase Spb40, followed by D-amino acid oxidase (DAO) homologous enzyme Spb39 catalyzing the intermediate in a FAD-dependent manner with the cleavage of the Cɑ–N bond and oxidizing to form hydrazinoacetic acid (HAA) and releases 2-aminoadipate 6-semialdehyde (AASA). The mechanism of subsequent s56-p1 N–N bond formation was unclear [53]. However, Some of the genes involved in s56-p1, azaserine and triacsins biosynthesis are homologous, and thus they are all converted to HYAA-PCP intermediates using L-lysine and L-glycine as precursors [12, 110].

In the biosynthesis of triacsins, Tri26 and Tri28 generate a lysine-glycine intermediate, which is processed by the DAO homolog Tri27 to generate HAA and AASA. HAA then undergo succinylation, ACP transfer, oxidation, chain extension and hydrolysis. Subsequently the *N*-hydroxytriazene pharmacophore is generated in the presence of Tri16/Tri21 and Tri17. Ultimately, triacsins can be generated by non-oxidative decarboxylation of the UbiD-family decarboxylase Tri10 and the UbiX homolog Tri9, as well as by the oxidative or reductive action of the putative Tri18/Tri19 [12, 111].

In the biosynthesis of azaserin, the enzymes AzsL, AzsM and AzsN are present that are homologous to the enzymes Sbp38, 39 and 40. These enzymes catalyzes the synthesis of HAA from L-lysine and glycine. The HAA is then formed into HDA-AzsN by a series of homologous enzymes of triacsin (AzsS/AzsT/AzsP/AzsF/AzsD), and HYAA-Ser is formed by the interaction of the three structural domains of AzsO. Finally, AzsG and AzsE are hypothesized to catalyze the dehydrogenation of HYAA-Ser to form azaserine [12, 112].

Van Cura et al. identified another biosynthetic gene cluster (*aza*) for the synthesis of azaserine, containing nine genes *azaABCEFGMOQ*. Of these, the predicted oxidoreductases AzaL and AzaN may be involved in the formation of the diazo group, but sufficiently relevant experimental validation is lacking [110].

### Formation of N–N bonds via nitric oxide and nitrosyl groups

The synthesis of *N*-nitroso-containing compounds, such as the *N*-nitroso of streptozocin, required the di-domain protein protein SznF/StzF [113, 114]. Its diiron domain first catalyzes the double hydroxylation reaction of *N*^ω^-monomethyl-*L*-arginine formed by StzE/SznE to generate *N*^δ^-hydroxy-*N*^ω^-hydroxy-*N*^ω^-methyl-L-arginine, followed by oxidative rearrangement of the *C*-terminal cupin domain to form *N*-nitroso-containing streptozocin [17].

Another gramibactin biosynthesis may be accomplished by enzymes encoded by the *grbD* of the *stzF/sznF* homologous gene and *grbE* gene. GrbE is hypothesized to catalyze the hydroxylation of* L*-arginine, and then GrbD may catalyze the formation of the N–N bond of *L*-graminine [17, 67, 115]. Subsequent involvement of *L*-graminine and other enzymes in the synthesis of graminibactin may continue [17, 67].

### Formation of N–N bonds through radical coupling

Dixiamycin is formed by coupling the N–N bond of xiamycin A. In vivo experiments, xiamycin A is incubated with *Streptomyces* sp. HKI0576 expressing the flavoenzyme XiaH (or XiaK) to obtain dixiamycin [116, 117]. In vitro experiments, XiaK hydroxylates xiamycin A to form *N*-hydroxyxiamycin. It is observed that *N*-hydroxyxiamycin formed a nitroxyl radical under aerobic conditions, and the researchers hypothesizes that the two nitroxyl radicals might dimerize to form dixiamycin by combining and removing an oxygen molecule [118]. The mechanism of dixiamycin formation is still not clear. It is also possible that dixiamycin is formed by the non-radical forms of xiamycin A and *N*-hydroxyxiamycin [17].

The enzymes involved in azoxy bond formation have also been studied in recent years. In the biosynthesis of azoxymycin, a nonheme diiron *N*-oxygenase AzoC could catalyze the direct oxidation of amine to nitroso group, and then the conversion between hydroxylamine and nitroso products could be nonenzymatically facilitated by the action of coenzymes such as NAD^+^/NADH. During the conversion process, it is found that the hydroxylamine and nitroso products could not be converted to azoxy products by introducing the typical radical scavengers 2,2,6,6-tetramethyl-1-piperidinyloxy (TEMPO) and butylated hydroxytoluene (BHT), proving that their respective free radical couplings could form azoxy bond [119].

### Additional enzymatic routes for N–N bond formation

The biosynthetic logic of constructing heterocyclic compounds containing N–N bonds differs from that of forming a hydrazine and other N–N linkage [120]. For example, pyrazomycin and formycins contain a rare *C*-nucleoside structure encoded by the *Prf* (*Streptomyces candidus* NRRL 3601) and *For* (*Nocardia interforma* ATCC 21072/ *Streptomyces kaniharaensis* SF-557) gene clusters, respectively [121]. Their biosynthetic pathways mainly consiste of two parts: before and after the critical *C*-glycosylation of the pyrazole intermediate DCOP [12]. The lysine *N*^6^-monooxygenase PrfK/ForK catalyzes the synthesis of *N*^6^-OH Lys from lysine, and then PrfJ/ForJ actes as a hydrazine synthase catalyzing the generation of the Lys-Glu intermediate from *N*^6^-OH Lys and L-glutamate. Multiple enzymes assemble DCOP and *C*-glycosylated by the β-RFA-P synthase-like enzyme PrfT/ForT. Downstream enzymes PrfCBFH and ForCBHAP/PurH synthesize pyrazomycin and formycins, respectively [121, 122].

The biosynthetic gene for the formation of N–N bond in piperazinic acid is ktzT [11]. First the *N*-hydroxylation of L-ornithine to form *N*–OH-L-ornithine is catalyzed by the gene KtzI encoding NMOs [123], and then the formation of the N–N bond is catalyzed by ktzT [11]. Meanwhile, a mononuclear iron halogenase KthP and a P450 monooxygenase HmtN catalyze the formation of chloropiperazic acid and hydroxylated piperazinic acid, respectively, which then undergoes other enzymes to form kutzneride [124] and himastatin [17, 125]. The cytochrome P450 Luz26 catalyzes the 1,6-dehydrogenation of piperazinic acid by hydroxylation followed by dehydration to form luzopeptin A [17, 126].

Actinopyridazinone is another class of structures containing a dihydropyridazinone heterocycle and the ring-forming mechanism has only recently been investigated [127]. After the previously described generation of DABA-Ala by Apy9, Nacetyl-2-amino-4-hydrazineylidenebutanoic acid (*N*ω-acetyl AHYBA) is produced in response to FAD-dependent cleavage of the C–N bond catalyzed by the oxidase Apy10 and *N*ω-acetylation by the *N*-acetyltransferase Apy2. The mechanism of dihydropyridazinone formation is based on the activation of *N*ω-acetyl AHYBA by Apy3, which is then loaded onto the Apy6 carrier domain. The successive removal of the acetyl group by Apy4 results in an intramolecular nucleophilic attack of the unprotected distal nitrogen on the carbonyl carbon, leading to the formation of dihydropyridazinone [127].

Another heterocyclic compound is toxoflavin, whose biosynthesis begins with the formation of the dephosphorylated intermediate by a GTP cyclohydrolase II ToxB, a bifunctional deaminase-reductase ToxE [128] and the alkaline phosphatase (CIP) treatment [129]. The next synthetic pathway is resolved in detail by Liu et al. Only *toxBE* and *toxBDE* are found to be incapable of accumulating two new compounds, i.e., 1,6-dedimethyltoxoflavin and 1-ribityl-dedimethyltoxoflavin. Using ^13^C-labeled GTP experiments and expression of *toxBCE* and *toxBCDE* in *E. coli* strains, it is verified that toxD may catalyze the conversion of 1-ribityl-dedimethyltoxoflavin to 1,6-dedimethyltoxoflavin [129]. ToxC is hypothesized to catalyze the reaction between glycine [130] and the dephosphorylated intermediate to produce 1-ribityl-dedimethyltoxoflavin [129]. Finally ToxA undergoes two *N*-methylation reactions to form toxoflavin [131].

**Fig. 9:**
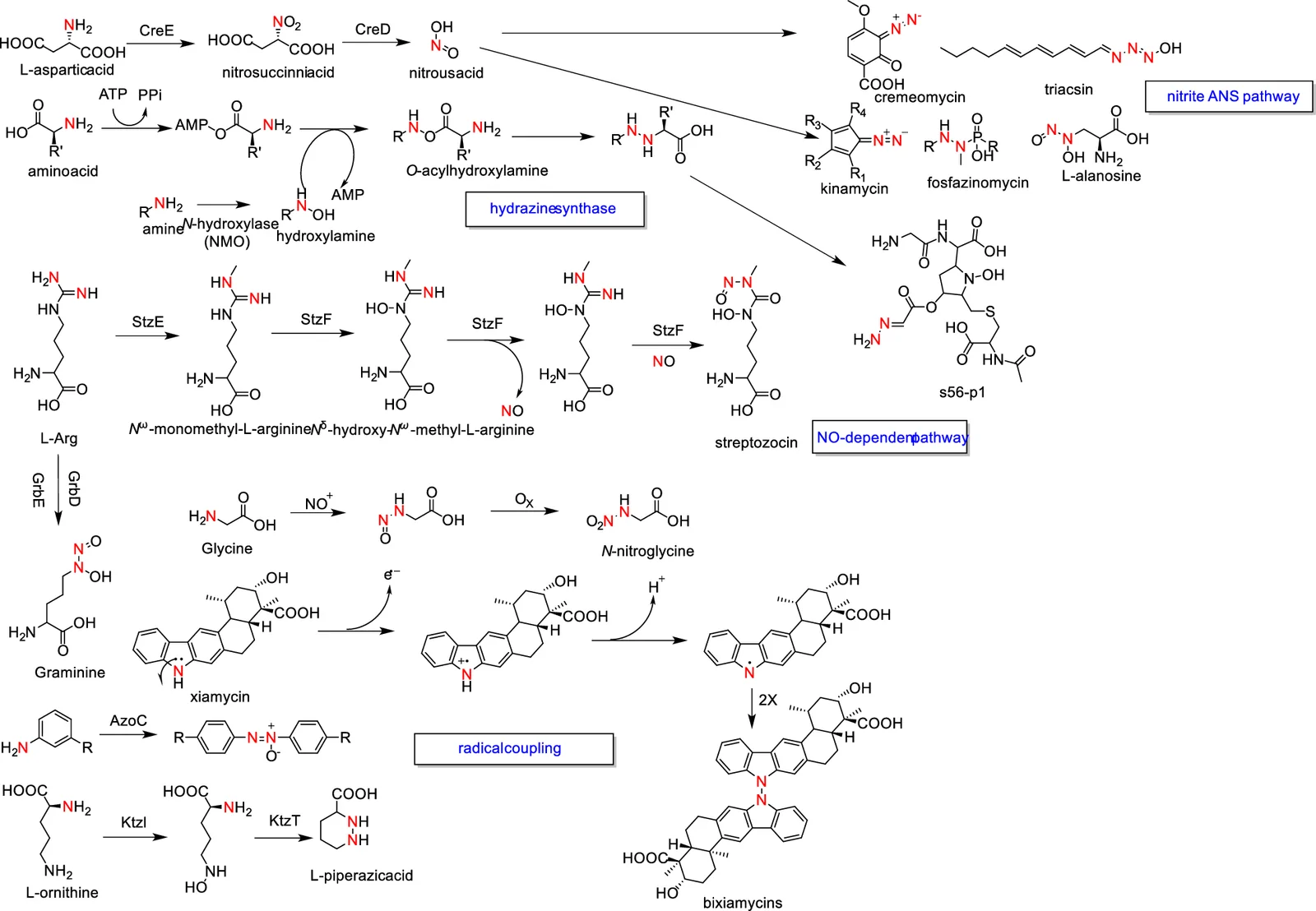
Diverse enzymatic pathways for the biosynthesis of natural products containing N–N bonds

8-Azaguanine is a triazole-containing heterocycle. Huang et al. proposed a biosynthetic pathway by genome sequencing, heterologous expression, and in vitro experiments [132]. GCH I homolog 8-Azg F catalyzed the generation of 2,5-diamino-6-ribosylamino-4(*3H*)-pyrimidinone 5′-triphosphate intermediate from GTP. NOS 8-AzgA yields NO from *L*-arginine, and 8-AzgB shuttles NO/N_2_O_3_. There may be two different pathways for this intermediate: direct reaction with reactive nitrogen species (NO) or a radical intermediate catalyzed by 8-AzgG, which then reactes with reactive nitrogen species and dephosphorylated to form 5′-monophosphate 8-azaguanosine or it undergoes dephosphorylation first. Alternatively, it forms 8-AzgD ultimately catalyzed the hydrolytic removal of 5′-monophosphate 8-azaguanosine to form 8-Azaguanine [132]. This pathway has similarities with the 8-Azaguanine biosynthetic pathway proposed by Zhao et al. [133] (Fig. 9; Table 1).

**Table 1:** Comparative summary of eight types of N–N bond-forming biosynthetic enzymes

Enzyme family	Catalytic substrate	N–N formation mode	Representative natural products
FAD-dependent monooxygenase (CreE) and nitrosuccinate lyase (CreD)	L-aspartic acid	ANS pathway	Cremeomycin
Hydrazine synthase	L-amino acids; *N*^ω^-hydroxylamine substrates	MetRS-like domains—hydroxylation of the substrate; zinc-binding cupin domain—hydrazine molecules	S56-p1; Triacsins
Diiron/cupin bifunctional protein (SznF/GrbD)	*N*^ω^-monomethyl-L-arginine	Diiron domain—double hydroxylation of arginine; cupin domain—oxidative rearrangement to form *N*-nitroso N–N linkage	Streptozocin; Gramibactin
Flavoenzyme (XiaH/XiaK); Nonheme diiron *N*-oxygenase (AzoC)	Xiamycin A (monomer); amines	Radical coupling	Dixiamycin; Azoxymycin
Lysine *N*_6_-monooxygenase (PrfK/ForK); hydrazine synthase (PrfJ/ForJ)	L-lysine; L-glutamate	PrfK—Lysine N-hydroxylation; PrfJ—Intermolecular N–N coupling	Pyrazomycin; Formycins
FAD-dependent N-monooxygenase (KtzI); heme-dependent piperazate synthase (KtzT)	L-ornithine	KtzI—N-hydroxylation of L-ornithine; KtzT—intramolecular cyclization to form piperazine acid	Verucopeptin; Caveamides; Kutzneride; Diperamycin
Hydrazine synthase (Apy9); FAD-dependent oxidase (Apy10)	*N*_4_-hydroxy-L-DABA	Apy9—generates a hydrazine intermediate(DABA-Ala); Apy10—breaks the C–N bond and forms a heterocycle via an intramolecular cyclization reaction	Actinopyridazinone
GTP cyclohydrolase II (ToxB); ToxC	Glycine; GTP-derived intermediate	ToxB—forms a dephosphorylated intermediate; ToxC—forms the triazole core	Toxoflavin
NO synthase (8-AzgA); NO transporter (8-AzgB)	L-arginine	8-AzgA—NO production; 8-AzgB—NO delivery for heterocyclic formation	8-Azaguanine

## Biosynthesis

### Valanimycin

Valanimycin (**a10**) was a natural azoxy antibiotic isolated from *Streptomyces viridifaciens* MG456-hF10 in 1986 whose core N–N linkage is constructed via hydrazine synthase machinery summarized in Sect. 4.2. It possessed antibacterial activity against certain Gram-positive and Gram-negative bacteria as well as toxicity against mouse leukemia cells [134]. Early precursor-feeding studies by Parry’s group confirmed L-valine (**a1**), serine and isobutylamine as key biosynthetic precursors [135, 136].

Gene-functional characterization reveales that flavin monooxygenase vlmH which is functionally similar to the NAD(P)H:FMN oxidoreductase of *Photobacterium fischeri* [137, 138] and flavin reductase vlmR [139] which is the closest match to the NADH:FMN oxidoreductase SnaC protein [140] work jointly to hydroxylate of isobutylamine (**a2**) into isobutyl hydroxylamine (**a3**) (IBHA) [139]. Notably, by synthesizing [^15^N,^18^O]-isobutyl hydroxylamine hydrochloride precursor incorporation experiments, it is found that the hydroxyl oxygen of isobutyl hydroxylamine is not the source of the azoxy-group oxygen. Although the researchers had proposed two mechanisms for the formation of azoxy group, further research is still needed [141].

Parry et al. clarified the scope of the valanimycin biosynthesis gene cluster and the functions of the associated genes [142]. The *vlmE* gene encodes a repressor protein that may bind to the promoter of the resistance gene *vlmF* and regulates the expression of the *vlmF* gene [142]. VlmD may encode a valine decarboxylase [142] and vlmL catalyzes L-serine (**a4**) to generate L-serine-tRNA (**a5**) in the ATP-dependent presence [143].

The researchers go on to explore the next route of synthesis of L-seryl-tRNA. Isobutyl hydroxylamine and L-[U-^14^C]seryl-tRNA are incubated with *S. viridifaciens* cell-free extracts and three radioactive products are found. Using vlmJ, vlmK and vlmA mutated *S. viridifaciens* prepare cell-free extracts are incubated with the same reactants as the first time and vlmH is used as a control, the experimental results shows that there is no difference between the cell-free extracts prepared from vlmJ, vlmK and vlmH mutants and the wild type respectively. While the extracts prepared with vlmA mutant do not produce new radioactive products. When purified vlmA is utilized to react with an excess of wild-type incubation reactants, it is found that *O*-(L-seryl)-isobutyl hydroxylamine catalyzed by vlmA is rearranged to *N*-(L-seryl)-isobutyl hydroxylamine (**a6**) within a certain time [144].

Subsequently, using [U-^14^C]-L-serine and L-valine with vlmJ and vlmK mutants of *S. viridifaciens* are incubated with washed cells, and valanimycin hydrate (**a7)** is found. Cell-free extract from the vlmJ mutant failes to convert this hydrate into valanimycin, implying that vlmJ participates in dehydration. By contrast, the vlmK mutant convertes the valanimycin hydrate into a phosphorylated product (**a9)**, but is unable to produce valanimycin. Based on the above experiments, vlmJ and vlmK catalyzes the phosphorylation and dehydration reactions of the valanimycin hydrate to generate valanimycin [145].

However, the enzymatic process from **a6** to **a8** and the formation of the N–N bond in the azoxy group has not been resolved until recently [146]. Through precursor incorporation experiments suggested that hydrazino acid generates from isobutyl hydroxylamine and serine could be involved in the synthesis as an intermediate [147]. One proposed mechanism states that **a6** first forms an intermediate **a8**, and then valanimycin hydrate containing an azoxy group was formed by four-electron oxidation [146].

Three uncharacterized genes remain in the valanimycin gene cluster: *vlmG*, *vlmB* and *vlmO*. Heterologous expression assay showes that vlmO catalyzes the conversion of *N*-(L-seryl)-isobutyl hydroxylamine into a hydrazine intermediate to form a N–N bond. VlmB showes that it encodes a non-heme diiron enzyme. VlmO-microsomes are used to catalyze the generation of **a8** from **a6**, and ultrafiltration is used to remove vlmO in vitro experiments. VlmB is then incubated with the deproteinized reaction product and other accessories. VlmB produces **a7** at low yield. Therefore, the synthetic hydrazine intermediate is used to react with vlmB again, and surprisingly, in addition to producing the azoxy product, a secondary isomeric azoxy product is also produced [146]. The use of Marfey’s analysis [148] suggestes that the stereochemical change at the C2 in the hydrazine intermediate may be responsible [146].

**Scheme 1:**
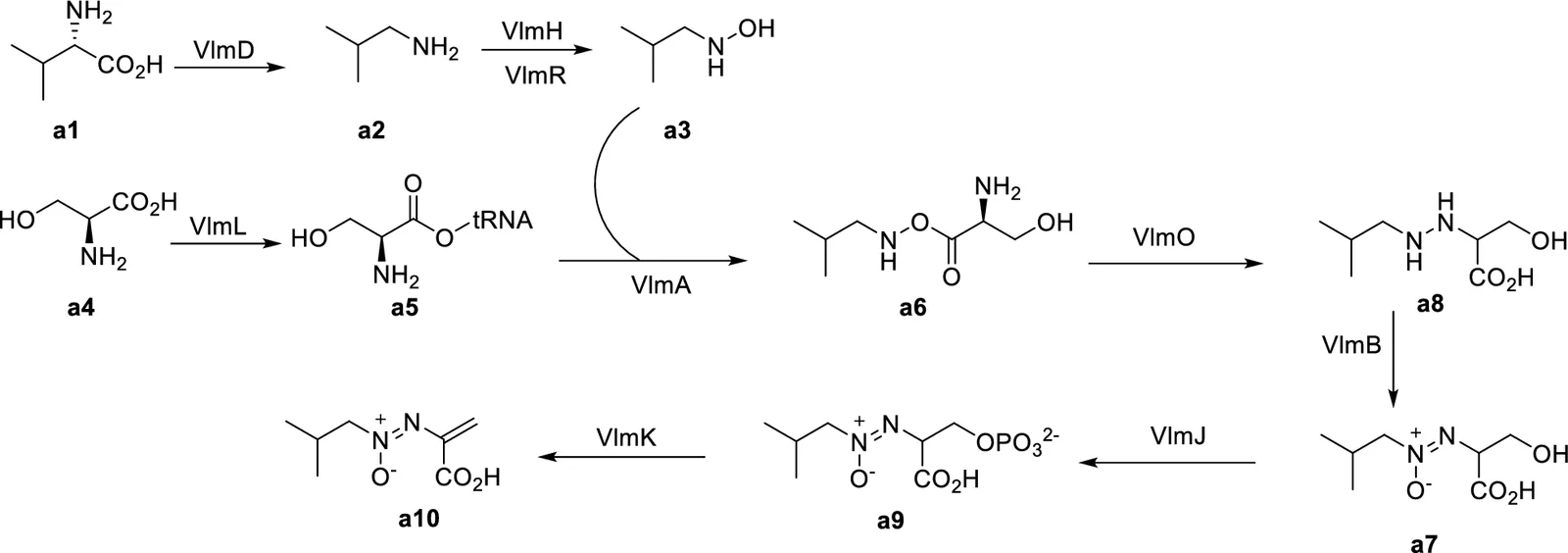
Biosynthesis of valanimycin (**a10**)

Labeling experiments with ^18^O_2_/H_2_^16^O and ^16^O_2_/H_2_^18^O clarifies that the oxygen atoms of the azoxy groups are derived from molecular oxygen. The researchers then elucidates the catalytic mechanism of vlmB by Mössbauer spectroscopy. The reduction of the diferric complex of vlmB by D-hydrazine intermediate produced the diferrous complex and the azo compound. The diferrous complex reduces oxygen and undergoes transformation to produce an electrophilic µ-1,1 hydroperoxo diferric complex and finally a nucleophilic attack by the azo compound to form the azoxy group. Alternatively, the N=N double bond double bond of the azo compound forms the oxadiazepine intermediate by oxygen atom transfer, which subsequently openes the ring to form the azoxy product (Scheme 1) [146].

### Spinamycin

Spinamycin (**b8**) was isolated from *Streptomyces albospinus* JCM3399 in 1966 [149, 150]. Two potential derivatives of spinamycin are also identified as 1-(3’-aminophenyl)-1,3,5-hexatriene and carboxylation products of spinamycin [151]. Its biosynthesis originates from the nitrite-dependent ANS biosynthetic pathway described in Sect. 4.1.

Because of the similarity between SpiV and the 3-ABA synthase PctV in pactamycin biosynthesis, **b2** is generated from SpiV-catalyzed 3-dehydroshikimate (3-DHS) (**b1**) [151]. Among them, SpiV synthesized **b2** using L-Glu as an amino group donor. The putative 3-deoxy-7-phosphoheptulonate synthase SpiF is responsible for providing the precursor for **b2** biosynthesis. To obtain the functions of the other enzymes predictes above for a more complete biosynthetic pathway for spinamycin, the researchers purifies these recombinant enzymes and performs in vitro experiments. SpiA3 substrate specificity experiments reveales that SpiA3 has a preference for 3-ABA [152] and that SpiA3 may have to bind to *holo*-SpiA4 in order to be active, loading 3-ABA onto *holo*-SpiA4, i.e. 3-ABA-SpiA4 **(b3)** [151].The ketoreductase (KR) spiA1 and SpiA5-SpiA6 heterodimers constitute a highly reducing (HR) type II PKS, and phylogenetic analysis indicates that the SpiA5-SpiA6 heterodimer is responsible for the synthesis of the triene portion of spinosyn [151, 153–155]. Therefore, SpiA5, SpiA6, SpiA1, and the presumed dehydratase (DH) of fatty acid synthetases generate 6-(3-aminophenyl)-2,4,6-heptatrienoyl **(b4)**, which is later hydrolyzed by an unknown enzyme to release it from SpiA4, in addition to the possible involvement of SpiB in the whole process [151].

Next, the researchers confirmed the involvement of *spi* clusters in spinamycin biosynthesis using a constructed *spi*ED gene deletion mutant strain. The *spi*ED gene deletion mutant strain is unable to produce spinamycin and its derivatives. Further expression and purification of the two domains of the SpiED in *Escherichia coli* BL21(DE3) and in vitro enzymatic experiments shows that SpiED is responsible for the generation of nitrous acid from L-Asp [151].

SpiA7 is hypothesized to catalyze the diazotization using **b4** as the substrate. Interestingly, cinnamic acid production is observed by incubating 3-aminocinnamic acid (3-ACA), an analog of 6-(3-aminophenyl)-2,4,6-heptatrienoic acid, with SpiA7, ATP, and MgCl_2_ and NO_2_^−^ and subsequent analysis of the Japp–Klingemann reaction [156] indicated that 3-diazocinnamic acid is the true product that cinnamic acid is produced because of destabilizing decomposition. Thus, SpiA7 catalyzes the diazotization reaction of **b4** and nitrous acid to produce 6-(3-diazophenyl)-2,4,6-heptatrienoic acid (**b5**) [151].

However, its relationship to the carboxylation product of spinamycin (**b6** and **b7**) is unknown [151]. Two hypotheses exist here, one was a SpiC-catalyzed four-electron reduction reaction of the diazo group and the DsbA family putative oxidoreductase [157] SpiC may be involved in the biosynthesis of the hydrazide moiety of spinamycin.The other involved a Japp–Klingemann-like reaction [151].

**Scheme 2:**
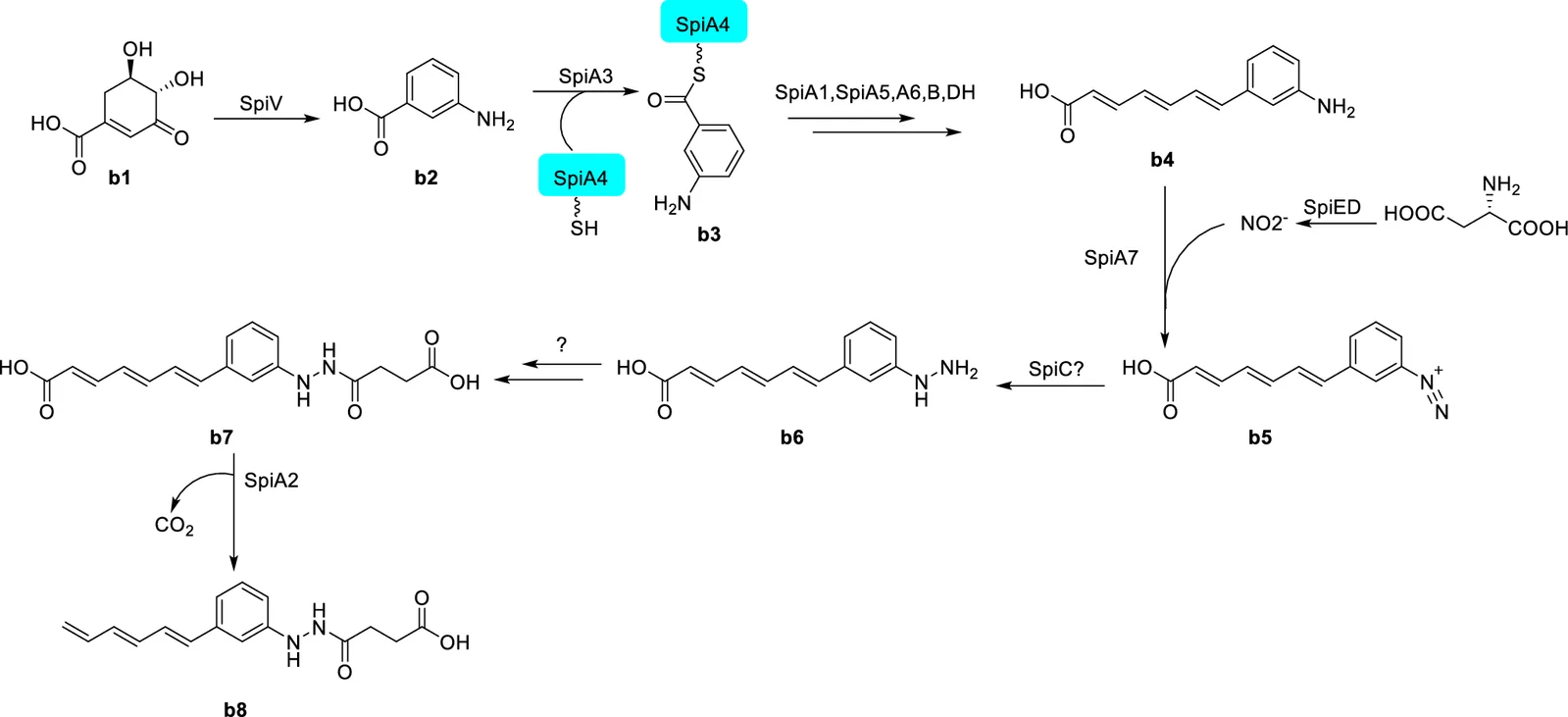
Biosynthesis of Spinamycin (**b8**)

In conclusion, the putative UbiD family decarboxylase [158] SpiA2 may act in catalyzing the terminal decarboxylation reaction of spinamycin biosynthesis [151]. The final step of SpiA2 catalyzes the terminal decarboxylation of the carboxylation product of spinamycin to synthesize spinamycin (Scheme 2) [151].

### Verucopeptin

Verucopeptin (**c16**), first isolated in 1993 from *Actinomadura verrucosospora* Q8862 [159, 160] and reisolated in the mangrove rhizosphere bacterium *Actinomadura* sp. XM-4-3 [161], is a piperazic acid-containing compound belonging to the pyranylated cyclodepsipeptides which matches the catalytic logic of piperazate-forming enzymes summarized in Sect. 4.5 [162].

The biosynthetic gene cluster of verucopeptin is composed of the typical three type I PKSs and four type I NRPSs, which containes thirteen PKS modules and six NRPS modules that assemble verucopeptin in the linear order of the type I modules. Meanwhile, VerP, VerN, VerL, VerO, VerM, and VerK synthesize the 2-methoxymalonic acid chain-expanding units (**c5**) in a synergistic manner by a 5-step stepwise reaction from **c1** to **c5**. The final PKS assembly formes the **c6** [161].

**Scheme 3:**
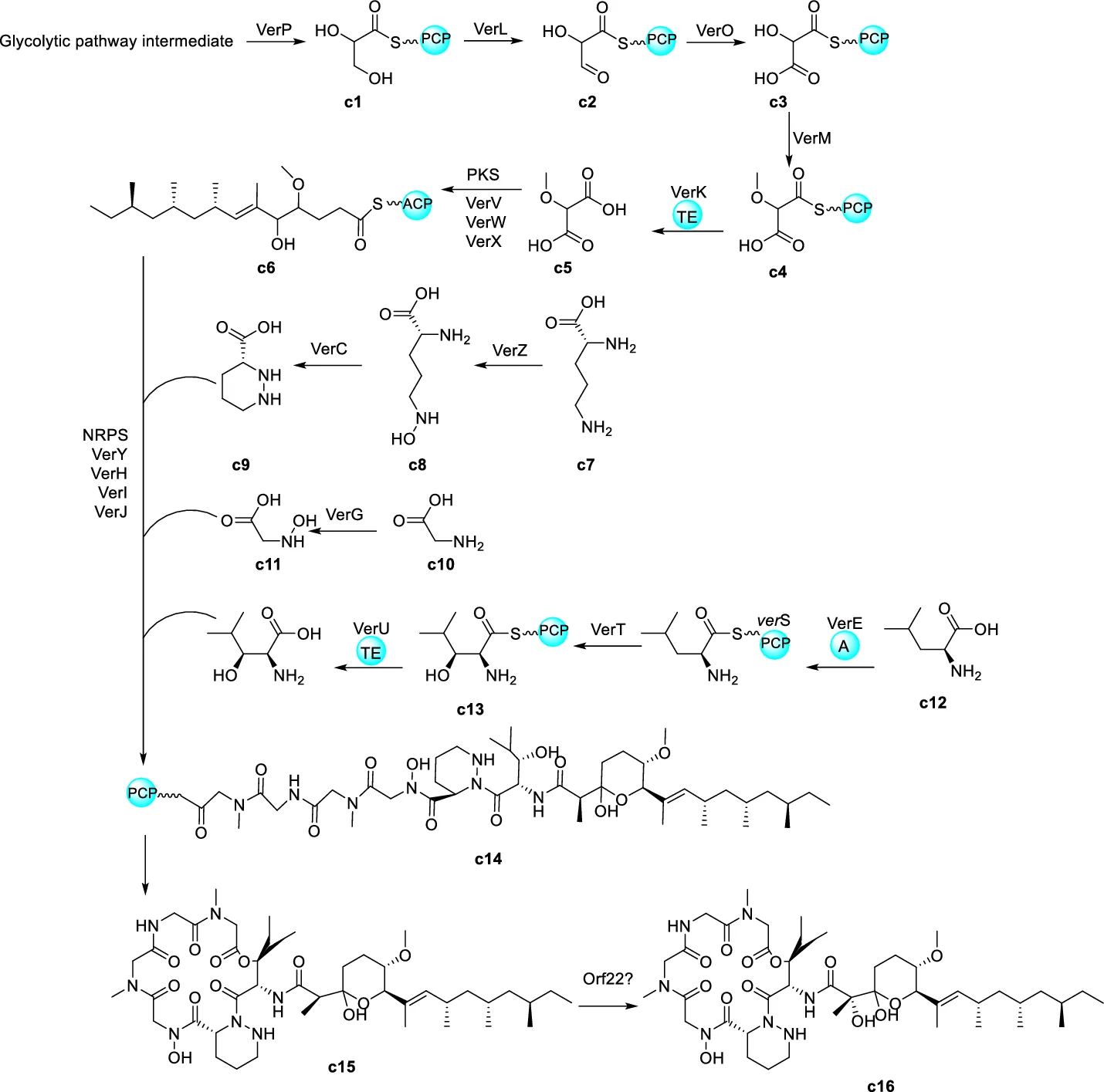
Biosynthesis of verucopeptin (**c16**)

While in the NRPS portion of the assembly, the researchers hypothesizes that VerZ catalyzed the first step of the ornithine (**c7)** hydroxylation reaction to produce **c8** and the heme-dependent enzyme VerC is predicted to catalyze the formation of piperazinic acid (**c9)** from **c8**. By mutating the *verG* and *verT* genes, it is found that VerG, encoding a hypothetical FAD-dependent oxidoreductase, are responsible for the amine hydroxylation of glycine (**c10)** to produce **c11**. VerT, a Cytochrome P450 monooxygenase gene, may be involved in the β-hydroxylation of leucine (**c12)** to produce **c13**, respectively. These enzymes, together with the type I module system, are involved in the assembly of verucopeptin [161]. The final NRPS assembly formes the **c14** and cyclize into **c15** [161]. The gene *orf22*, encoding a FAD-dependent oxidoreductase, is hypothesized to be involved in the last step of verucopeptin synthesis (Scheme 3) [161].

### Diperamycin

Diperamycin also incorporates piperazic acid building blocks, which shares the identical N–N bond construction mechanism of piperazate synthases described in Sect. 4.5. Malmierca team first screened the genomes of twelve sequenced “CS” strains with BLAST using KtzT as a probe [11, 45], and found a homologous protein of KtzT in *Streptomyces* sp. CS113, and its coding gene cluster was named *dpn* gene cluster [44].

The gene cluster is bounded by *dpnL* and *dpnR3* and contains genes for NRPS (*dpnS1*~*dpnS4*) consisting of six modules (M5–M10) and for type I PKS (*dpnP1*~*dpnP4*) consisting of four modules. By constructing a mutant of the non-ribosomal peptide synthetases (NRPS) gene *dpnS2* and culturing it together with the wild type CS113R in MS medium. Researchers analyzes and find that the mutants lacks a dipentomycin (DPN). During the biosynthesis of DPN, the *dpnO2* and *dpnZ* genes are hypothesized to encode ornithine monooxygenase and piperazate synthase, respectively, which synthesize the piperazic acid structure. The speculation is shown to be correct by constructing *dpnO2* and *dpnZ* mutant strains and backfilling them. The process of synthesis begins with the involvement of *DpnP1*–*DpnP4* in the synthesis of the polyketide chain, followed by the synthesis of the DPN peptide chain [44].

The AT domain of DpnP1 recognize methyl-malonyl-Coenzyme A [163] and transfered it to the ACP domain, which is then decarboxylated by the ketosynthase-like decarboxylase domain (KS_Q_) to form the initiator propionyl-ACP (**d1**) [44, 164]. The AT domain of DpnP2 recognizes hexylmalonyl-CoA and is extended following similar steps as DpnP1 to give **d2** [44, 163, 165, 166]. The subsequent DpnP3 and DpnP4 continue the polyketide chain extension. It should be noted that DpnP3 also contained a KR domain, and after reduction of the keto group to a hydroxyl group, and its DH domain underwent dehydration [44]. Ultimately a 2R product (**d3**) is formed by further reduction of the ER domain [44, 166, 167]. The final extension of DpnP4 forms a C15 polyketide chain **d4** [44].

DpnS1 to DpnS4 contains condensation (C), adenylation (A), and peptidyl carrier protein (PCP) domains [44]. The C domain of DpnS1 catalyzes the acylation of the threonine-PCP and C15 polyketide chains of the peptide chain to form **d5**. Then the A domain of M6 in DpnS2 recognizes L-piperazinic acid [168, 169] and transfers it to the PCP domain, the C domain catalyzes the condensation of L-piperazinic acid with threonine, and the E domain is responsible for the conversion of L-piperazinic acid to the D-configuration [44]. Next, the A domain of M7 are responsible for recognition and transfer it to the PCP domain and the MT domain further catalyzes the *O*-methylation of *N*-hydroxy-L-serine [44]. The C domain condense the methylated *N*-hydroxy-L-serine with D-piperazinic acid to ultimately obtain **d6** [44]. M8 and M9 and M10 are similar to the previous steps, with their A domains recognizing L-glycine, L-piperazinic acid and *N*-hydroxy-L-alanine, respectively. Their C domains then undergoes sequential condensation reactions to give **d7**, **d8**, and finally the entire polyketide-peptide chain product **d9** is released by cyclization of the TE domain of M10 [44].

**Scheme 4:**
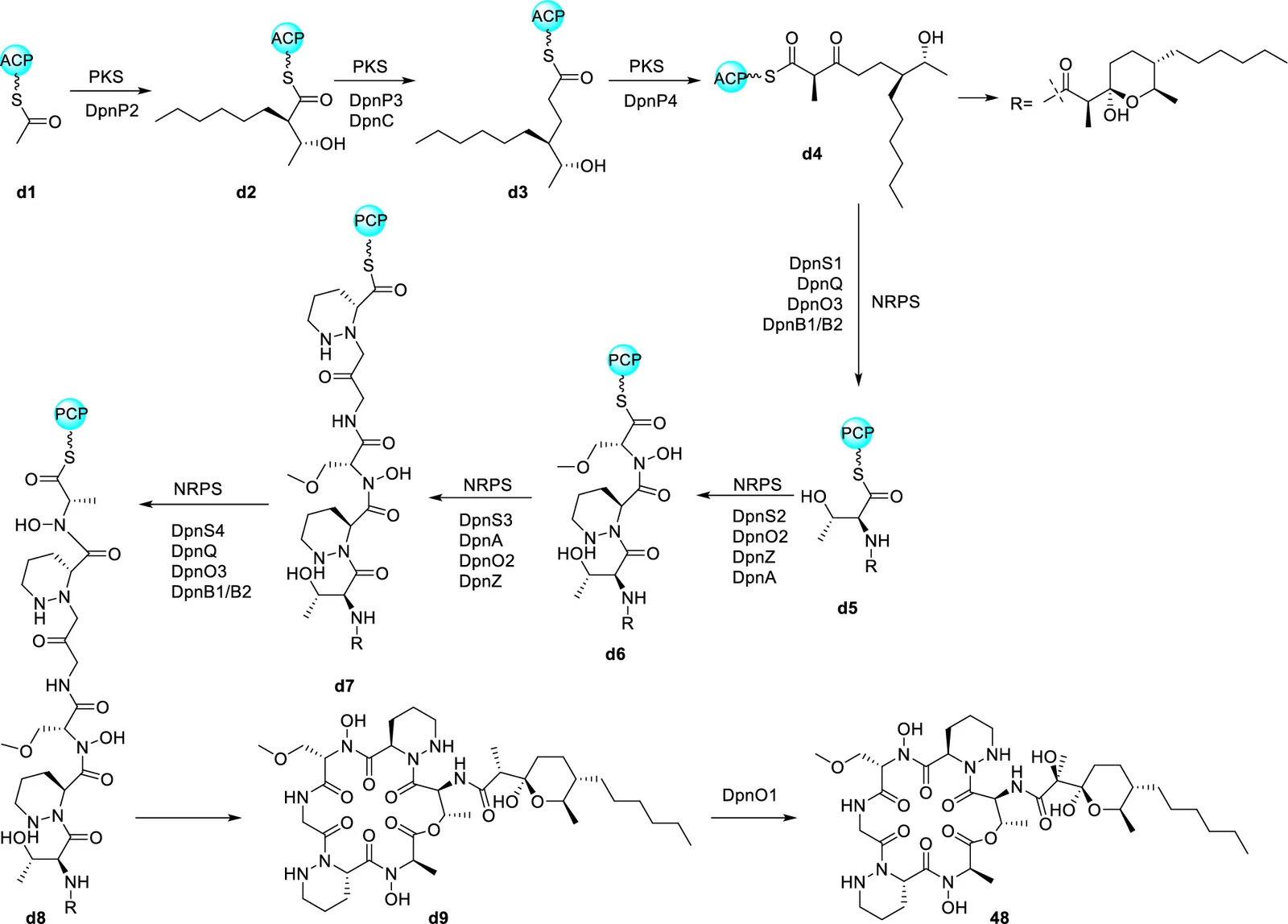
Biosynthesis of Diperamycin (**48**)

The final step may be hydroxylation to DPN by DpnO1, encoding a FAD-dependent monooxygenase [44]. One of them, *N*-hydroxy-L-alanine/*N*-hydroxy-L-serine may be accomplished by DpnO3 and the dissociated independent gene encoding NRPS DpnQ. DpnQ recognizes L-alanine/L-serine and transfers it to the PCP domain, which is then catalyzed by DpnO3 for its hydroxylation. Ultimately, DpnB1 and DpnB2 release the hydroxylation products to participate in DPN assembly (Scheme 4) [44].

### Future biosynthetic research directions

Despite the partially clarifies biosynthetic logic of valanimycin, spinamycin, verucopeptin and diperamycin, multiple critical intermediate conversion steps and catalytic mechanisms remain uncharacterized.

For valanimycin, the coordination mechanism underlying the cascade catalysis of VlmO and VlmB to form azoxy oxygen atoms remains unclear, and the function of the uncharacterized vlmG gene has not yet been confirmed. There is also currently no clear mechanistic explanation for the low catalytic yield of VlmB in vitro and the formation of stereoisomeric azoxy byproducts. In spinamycin biosynthesis, the link between diazo intermediate and spinamycin carboxylate derivatives is unvalidated, and two proposed hydrazide formation pathways lack biochemical proof. As for verucopeptin, the sequence of events in the modification of the piperazine acid moiety and the FAD-dependent oxidoreductase Orf22 have not yet been elucidated. The final step of the synthesis remains unresolved. For diperamycin, the function of dpnO3 has not yet been experimentally verified in the final step of *N*-hydroxyamino acid synthesis.

Therefore, in terms of future research directions in biosynthesis, validation of uncharacterized genes (VlmG, Orf22, DpnO3) in the target BGC through gene knockout and heterologous expression. Second, stable isotope labeling (^15^N-NMR) and liquid chromatography-mass spectrometry (LC–MS) techniques are used to identify unknown synthetic intermediates. Third, reconstruct incomplete catalytic cascades through in vitro enzyme recombination to determine the sequence of reactions involving the relevant enzymes. In addition, protein engineering techniques and Mössbauer/UV–vis spectroscopy can be used to elucidate the catalytic mechanisms of key oxidases [146].

## Chemical synthesis

The rich variety of compounds containing N–N bonding functional groups as well as the fact that most of them have better biological activities have caused scientists to study the chemical synthesis of such compounds. For example, Du’s team in 2022 summarized about the synthetic methods and pathways of functional groups [17]. Although some of these compounds have been synthesized, the synthesis of many N–N bond-containing functional groups still presents great challenges. This section summarizes several methods for the total synthesis of compounds.

### JBIR-39

JBIR-39 was a linear hexapeptide isolated in 2011 from *Streptomyces* sp. Sp080513SC-24 [170], but the *cis*-Piz(γ-OH) conformation has not yet been determined [171]. JBIR-39 has a structurally similar cyclohexadepsipeptide piperidamycins, but only its planar structure is known. The authors hypothesize that JBIR-39 is a precursor of piperidamycins and use the total synthesis of JBIR-39 to determine the structure–activity relationships between the two as well as its unknown configuration [171].

**Scheme 5:**
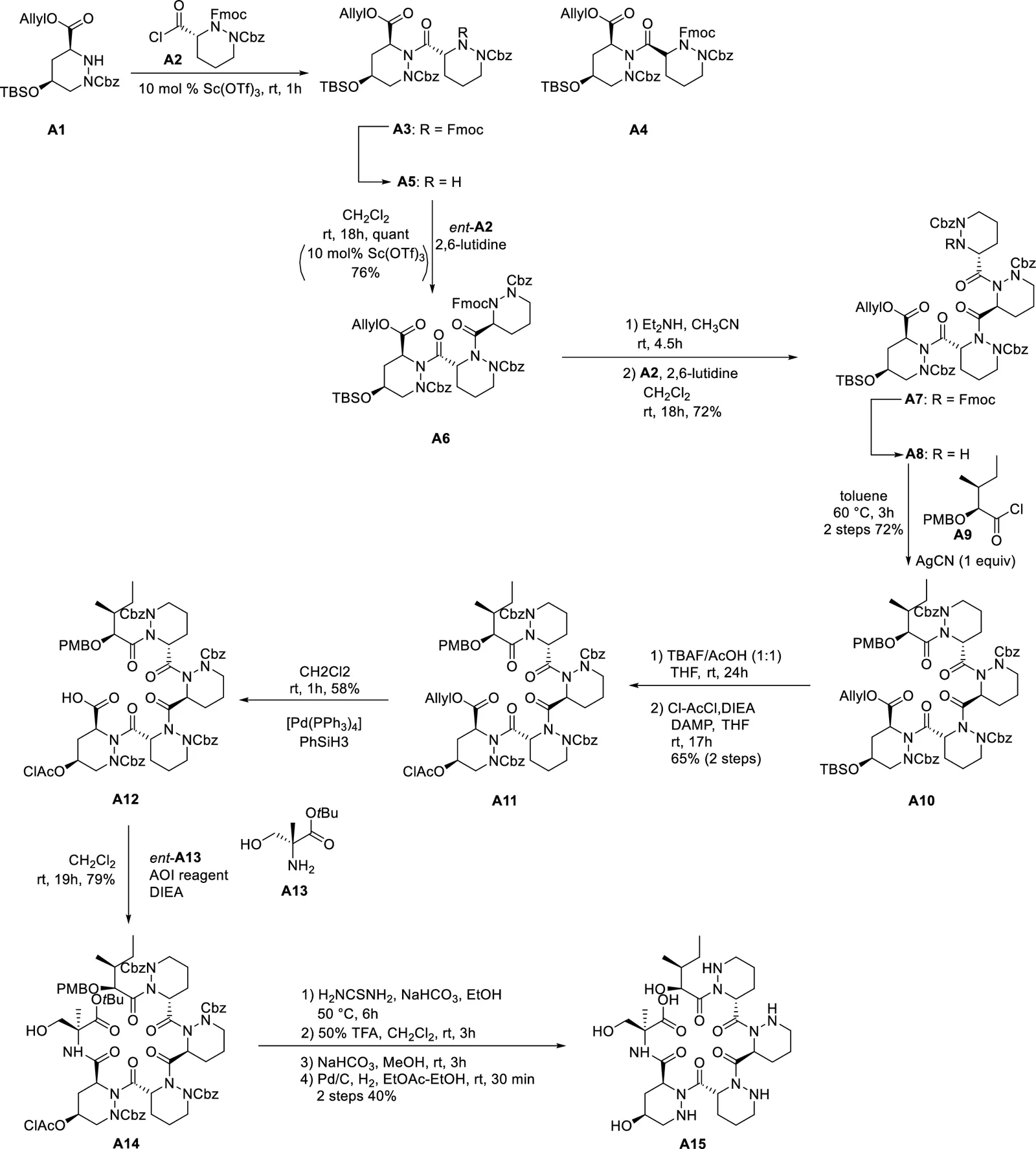
Chemical synthesis of JBIR-39 (**A15**)

The first total synthesis of JBIR-39 was accomplished by Doi et al. using the strategy of direct acylation of a H-Piz(γ-OH) derivative with hindered acid chlorides catalyzed by scandium trifluoromethanesulfonate (Sc(OTf)_3_). The core dipeptide structure Piz-Piz(γ-OTBS) derivative **A3** in 86% yield and its epimer **A4** is obtained by acylation catalyzed by Sc(OTf)_3_ using H-Piz(Cbz)(γ-OTBS)OAllyl (**A1)** and Fmoc-Piz(Cbz)Cl (**A2)** as substrates, followed by removal of the Fmoc moiety (**A5)** and the acylation reaction with *ent*-**A2** to obtain tri-piperazic acid derivative (**A6**). It then undergoes the same acylation reaction with **A2** to give tetra-piperazic acid derivative (**A7)** in 72% yield. Removal of the Fmoc moiety from **A7** gave **A8**, which is then further amidated in the presence of AgCN and l-isoleucyl chloride (**A9**) to give pentapeptide (**A10**) in 72% yield. The **A10** is then further converted to Piz (γ-OClAc) as a protecting group to give **A11** and its allyl ester to the corresponding acid **A12**. Then **A12** is amidated with (*S*)-*ɑ*-methylserine derivative *ent*-**A13** by AOI reagent to produce hexapeptide (**A14**) in 45% yield. The final deprotecting group gives (2*S*,6*S*,8*S*)-JBIR-39 (**A15**) in 40% yield, which is conformationally identical to the natural product (Scheme 5) [171].

### Prolinimines A–D

Prolinimines A–D were isolated from *Trichoderma* sp. CMB-F563, as well as a class of natural hydrazine compounds containing a rare Schiff bases. The first total synthesis of prolinimines A–D was accomplished by Capon and collaborators in 2018, solving the problem of the structural conformation of prolinimines D.

**Scheme 6:**
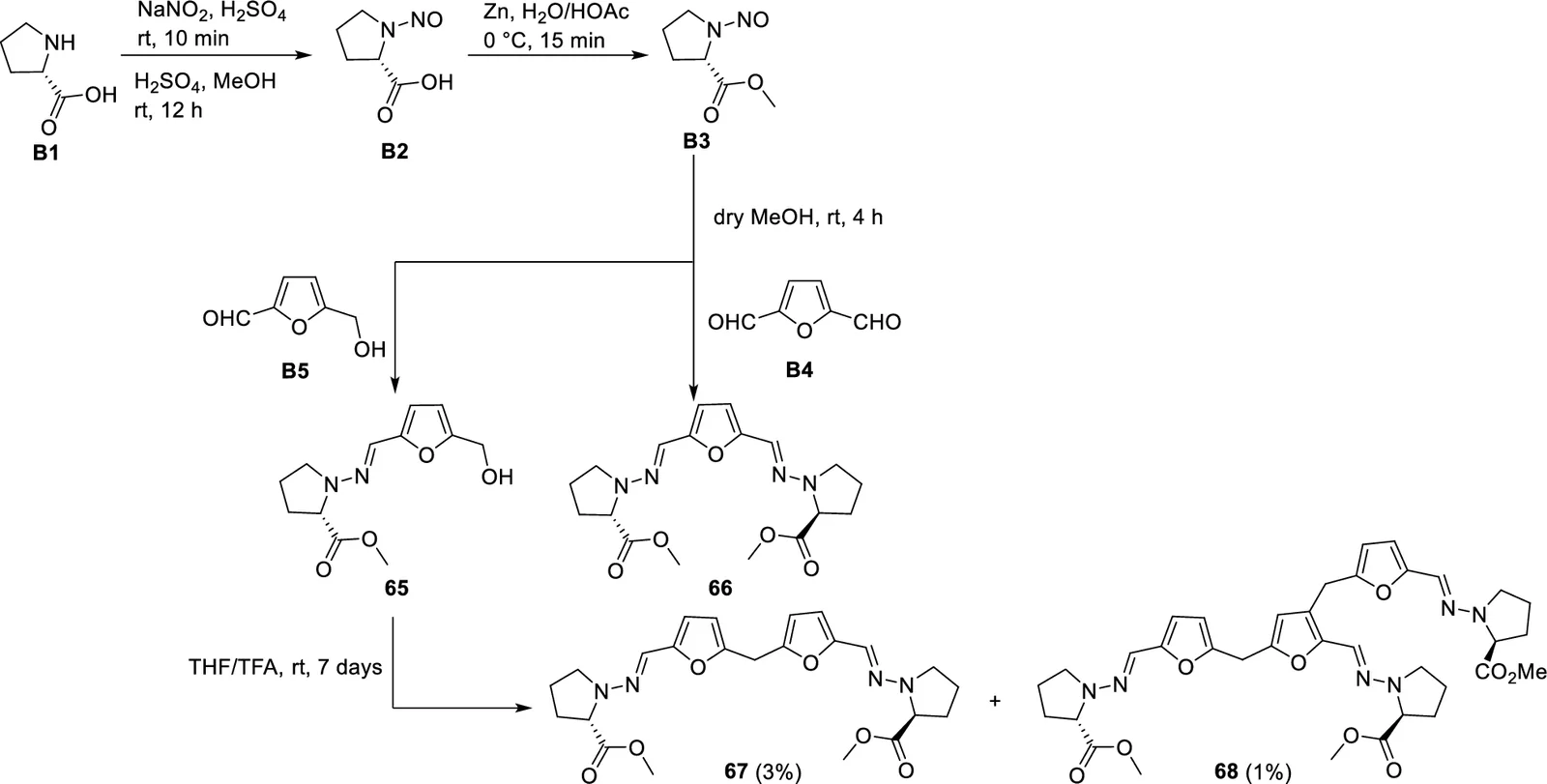
Chemical synthesis of Prolinimines A–D (**65–68**)

The synthesis starts with the readily available L-Pro (**B1**), which undergoes a two-step reaction to give its *N*-nitroso methyl ester (**B2**) and is further reduced to the important intermediate **B3**. Then **B3** undergoes a mono/double condensation reaction with 2,5-furandicarboxaldehyde (**B4**) or 5-hydroxymethylfurfural (**B5**), respectively, resulting in bis-Schiff base prolinimine B and mono-Schiff base prolinimine A. Prolinimine A produces prolinimines C and D under slightly acidic conditions (Scheme 6) [55].

### L-156,373

L-156,373 was isolated from *Streptomyces silvensis* in 1989 and was a potent oxytocin receptor antagonist [172]. Thus its activity has given rise to numerous chemists to study its medicinal chemistry as well as semisynthetic modification [173]. However, the presence of a specific Piz-Piz dipeptide subunit in the structure increased the difficulty of synthesis, and its first total synthesis was accomplished by Del Valle and coworkers using a sequential electrophilic amination-acylation-deprotection strategy.

The synthetic pathway begins with Glutamic acid benzyl esters (**C1**) and is followed by a four-step reaction including Fmoc-protection and reduction reaction with anhydride derivatives, silyl protection and hydrogenolysis to obtain orthogonally protected monomers (**C2**) in 65% yield. Then the (*N*–OH)-Ile building block (**C5**) in 66% yield is then obtained by diazotization-alkylation reaction to produce **C4** using H-D-*allo*-Ile-OH (**C3**) as substrate, followed by Mitsunobu reaction and PMB ester bond breaking reaction.

**Scheme 7:**
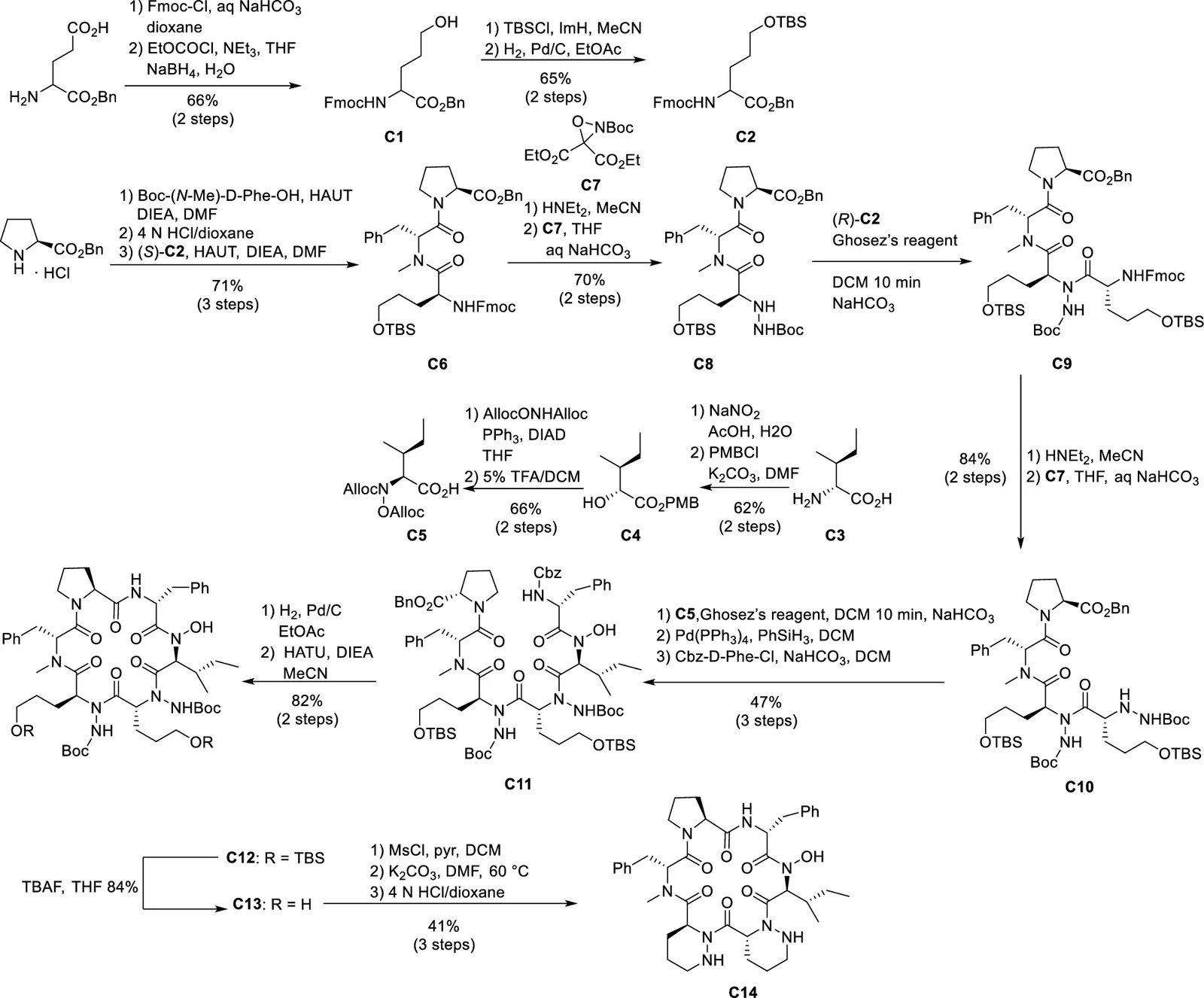
Chemical synthesis of L-156,373 (**C14**)

Cyclic peptide synthesis is started next. The tripeptide (**C6**) is synthesized by a HATU-mediated coupling reaction in 71% yield for the three-step reaction. The **C6** is then subjected to an *N*-amino reaction by oxaziridine (**C7**) after removal of the protecting group to give **C8** [174, 175]. It then undergoes a coupling reaction with the **C2** preactivated with Ghosez’s reagent to produce a tetrapeptide (**C9**). The **C9** undergoes a reaction similar to that at **C6** in the previous step to yield **C10**, and a chemoselective *N*-acylation reaction to give the pentapeptide (**C11**) in 47% yield over three steps. Subsequent two-step macrocyclization by HATU/DIEA gives the important precursor hexapeptide (**C12**) in 82% yield. Silyl ether cleavage then yields **C13** followed by cyclization and removal of the Boc group to give L-156,373 (**C14**) in 41% yield (Scheme 7) [176].

### Verucopeptin

Verucopeptin was a new class of HIF-1 inhibitors [177], and therefore its total synthesis plays an important role in its development in clinical therapy. Previously, only the cyclododecapeptide core of verucopeptin was synthesized by Cai and coworkers [178], and the total synthesis of verucopeptin was first accomplished by Kakeya and coworkers through a reverse synthesis strategy [178].

Alcohol (**D2**) is produced from readily available monoacetate (**D1**) starting material via a 6 steps reaction. Next, the **D2** undergoes a 6-step reaction including oxidation and Wittig reactions, reduction and oxidation reactions, the Evans aldol reaction and removal of the chiral auxiliary to give the diol (**D3**) in a yield of 55%. The **D3** is next protected with *tert*-butyldimethylsilyl (TBS) and subjectes to Mitsunobu reaction to give **D4**, followed by methanol de-esterification and addition of TBS protecting group to obtain a di-silylated compound **D5**. Finally, the di-silylated compound is subjected to selective deprotection and Dess-Martin periodinane (DMP) oxidation to obtain the key alkyne (**D6**) [164].

The synthesis of another important precursor aldehyde is started next. Another known diol **D7** [179] is synthesized by *p*-methoxybenzylidene protection of the diol to obtain **D8**, and then PMB deprotection followed by TBS protection of the hydroxyl group to synthesize the *O*-silylated **D9**. The **D9** is then reduced with DIBAL-H to yield **D10**, which is subsequently oxidized to yield **D11**. Finally, the aldehyde (**D12**) is obtained by oxidizing dimethyl acetal and removing the TBS moiety. Then **D12** and **D6** undergo lithium hexamethyldisilazide (LHMDS) addition reaction as well as oxidation and hydrogenation to obtain ketone (**D13**). The ketone then undergoes dimethyl acetal deprotection to give **D14** and Pinnick oxidation to produce carboxylic acid (**D15**) [180].

The synthetic pathway to the peptide core is carried out next. The synthesis is initiated in the Fmoc-glycine loaded Wang resin (**D16**). Fmoc sarcosine (**D17**) and *N*-hydroxy glycine (**D19**) are gradually introduced to form a tripeptide (**D20**). Among them, the synthesis of **D19** begins with Fmoc protection of the amino group of *tert*-butyl ester (**D18**) and is completed by removing the *tert*-butyl group under acidic conditions [180].

**Scheme 8:**
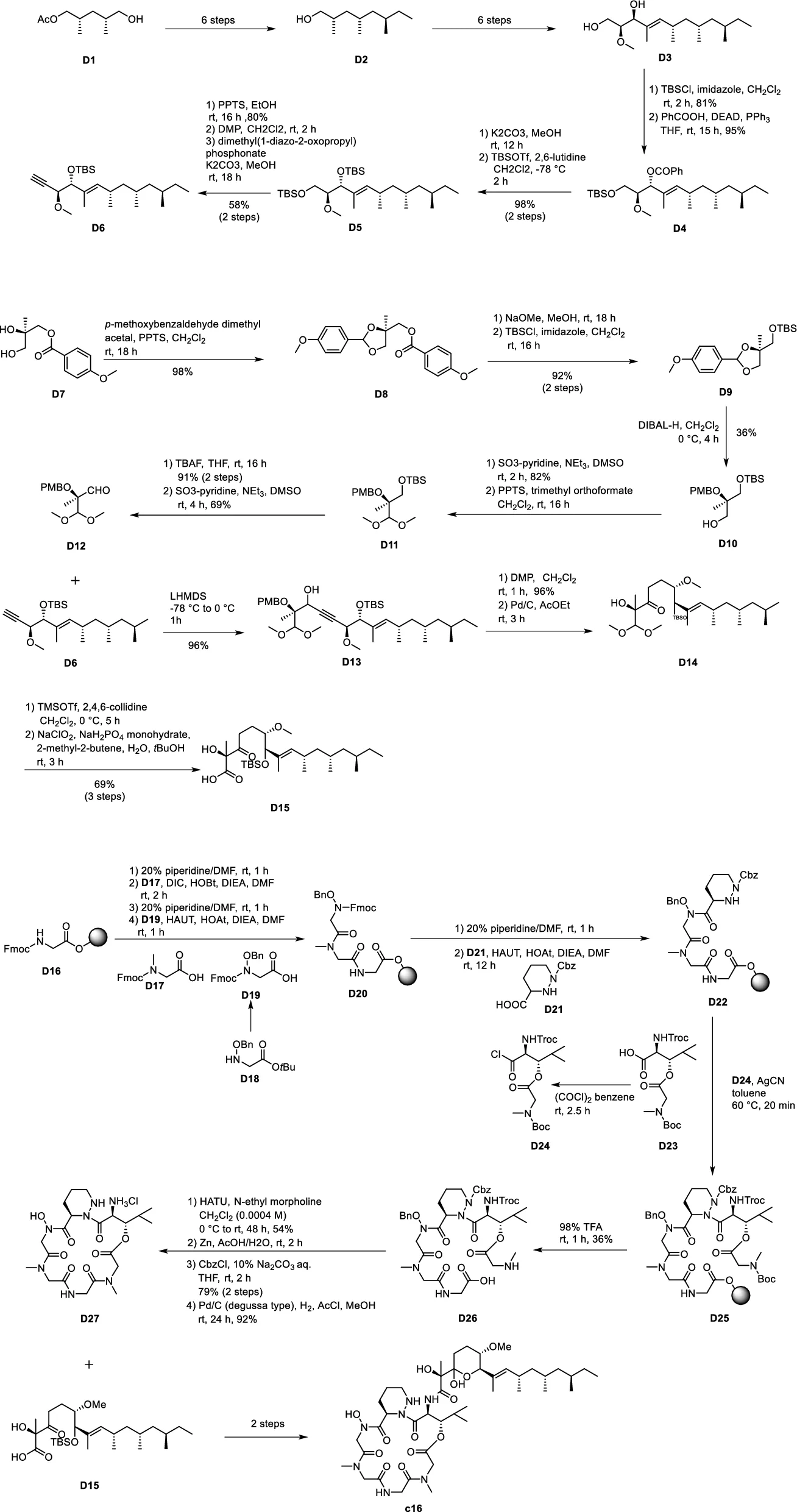
Chemical synthesis of verucopeptin (**c16**)

Then the piperazinic acid (**D21**) [181] is introduced to carry out the condensation reaction to obtain a tetrapeptide (**D22**). Next the dipeptide (**D23**) [178] is further introduced and converts to the acid chloride form (**D24**) [178, 182] to obtain a hexapeptide (**D25**) by coupling it with the **D22**. The product is detached from the resin and purified to give a linear peptide (**D26**) in 36% yield and converts to a peptide core (**D27**) according to Hale's method [178]. Finally, the peptide core is coupled with the side chain unit carboxylic acid (**D15**) to synthesize verucopeptin (**c16**) in 23% yield via a two-step reaction (Scheme 8) [180].

### Hytramycin V

Hytramycin V was isolated from *Streptomyces hygroscopicus* ECUM 14046 and similar to the other antituberculosis drugs capreomycin and streptomycin [183], exhibited antimycobacterial activity against *Mycobacterium tuberculosis* under hypoxic conditions as well as antimycobacterial activity against Grampositive bacteria [184]. Kigoshi and coworkers had performed the first total synthesis of Hytramycin V by a retrosynthetic analytical strategy [184].

The synthesis of H-Leu-OPMB (**E2**) is carried out from Fmoc-Leu-OH (**E1**) by a two-step reaction of PMB protection and removal of the Fmoc group. Then, starting from the readily available acyl oxazolidinone (**E3**), the intermediate **E4** is obtained through Hale's method [185]. After five steps of reaction, the piperacic acid derivative (**E5**) is obtained in 43% yield, and the enantiomer of **E5** are prepared [184].

**Scheme 9:**
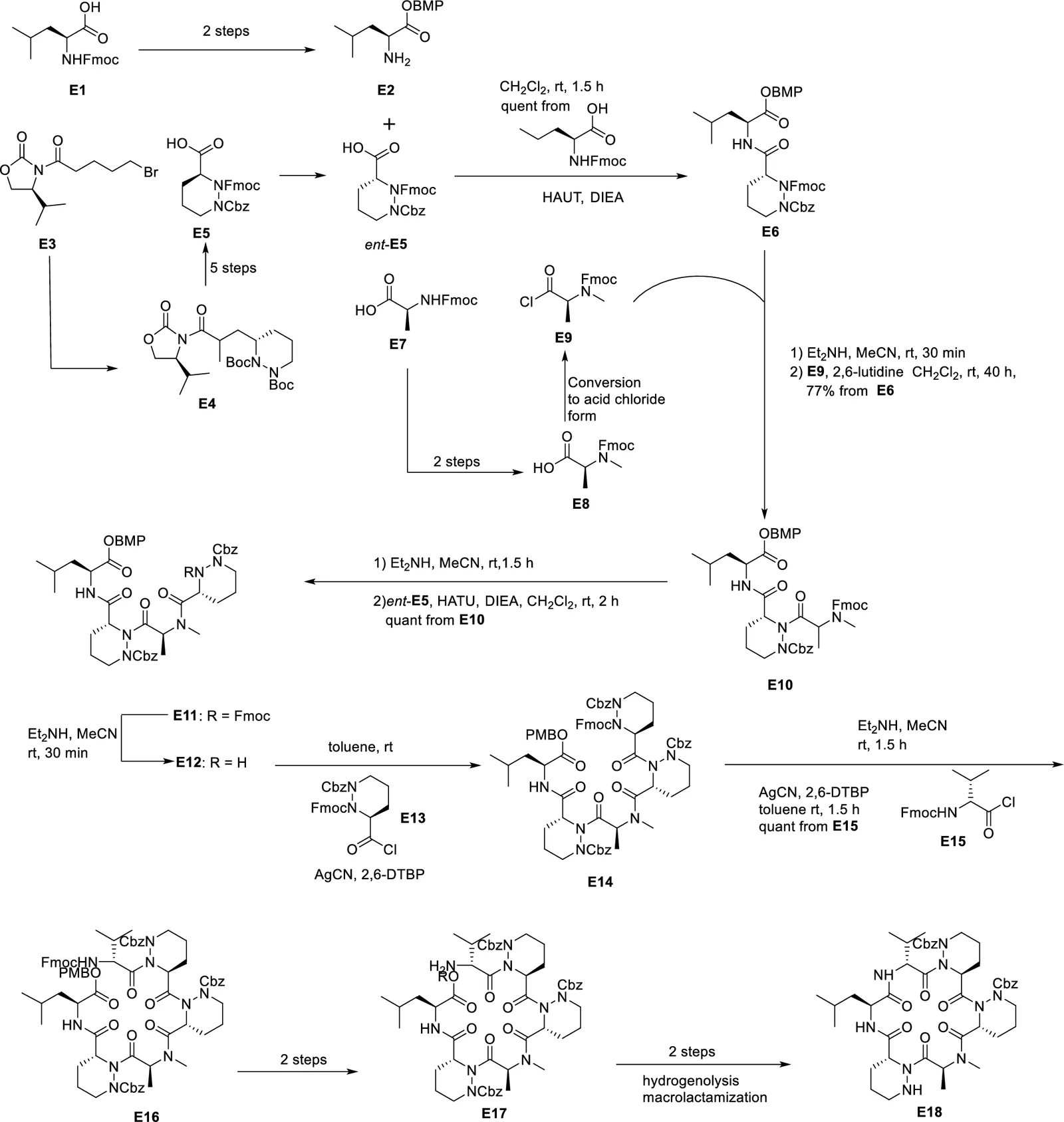
Chemical synthesis of Hytramycin V (**221**)

Cyclic peptide synthesis is initiated next. The first step is to generate dipeptides (**E6**) by coupling **E2** with the *ent*-**E5** via HATU/*N*,*N*-diisopropylethylamine (DIEA) [186]. Fmoc-MeAla-OH (**E8**) is obtained from Fmoc-Ala-OH (**E7**) in 89% yield via a two-step reaction [187]. Then amination with Fmoc-MeAla-Cl (**E9**) [188] is carried out to obtain the tripeptide (**E10**) in 77% yield after a two-step reaction. The **E10** is then coupled with the *ent*-**E5** via HATU/DIEA to generate a tetrapeptide (**E11**) in a two-step reaction. Next, the Fmoc protecting group of **E11** is stripped to form an amine **E12** which is then acylated with an acid chloride **E13** to give a pentapeptide (**E14**). The acylation is then continued with an acid chloride **E15** to obtain a hexapeptide (**E16**). The **E16** undergoes a two-step reaction to remove the C/N-terminal protecting groups to produce a cyclization precursor (**E17**). Finally, the hytramycin V (**E18**) is successfully synthesized by hydrogenolysis and macrolactamization (Scheme 9) [184].

### Pargamicin A

Pargamicin A (PrgA), isolated from *Amycolatopsis* sp, was a novel class of cyclic hexapeptide compounds that are effective against methicillin-resistant *Staphylococcus aureus* (MRSA) and vancomycin-resistant *Enterococci* (VRE) with strong antimicrobial activity associated with loss of membrane function and membrane depolarization [189, 190 ]. The difficulties in synthesis are increased by the single candidate site for macrolactamization of pargamicin A and the large steric bulk faces when incorporating with *N*ε-protected Piz residues, as well as the induced erosion of N-2 nucleophilicity by the carboxyl group may greatly exacerbate the stereoelectronic problem [182, 191, 192].

The important precursor substances are first prepared. The intermediate γ-OH-D-Piz (**F2**) is obtained by diastereoselective Cγ electrophilic hydroxylation of Troc-D-Glu(Bn)-OMe (**F1**) via a five-step reaction. Then further electrophilic amination is carried out to obtain building block **F3** in 73% yield over a two-step reaction [193].

**Scheme 10:**
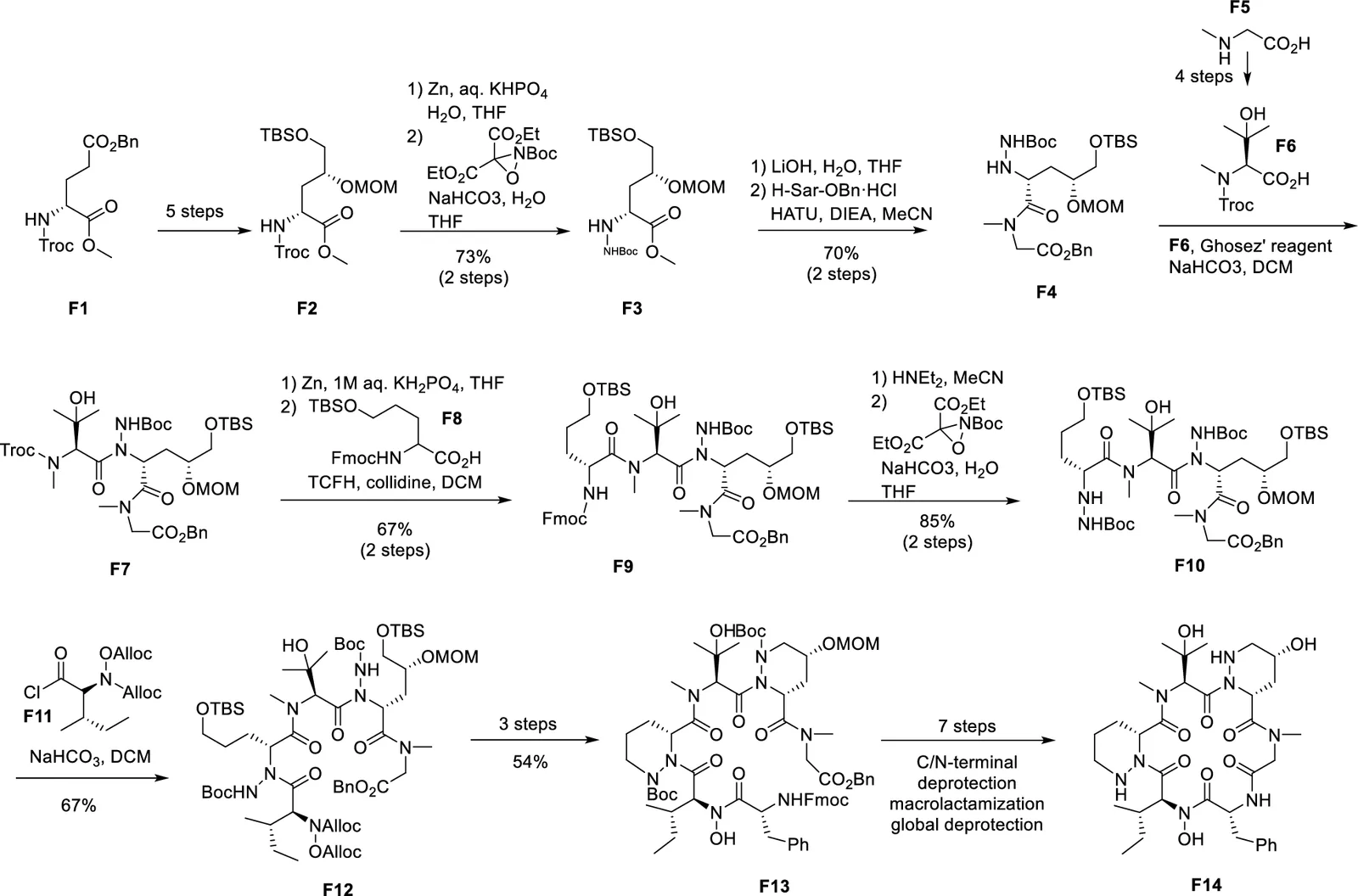
Chemical synthesis of Pargamicin A (**F14**)

Then the assembly of linear hexapeptide precursor is carried out. The **F3** is subjected to methyl ester hydrolysis and HATU-mediated condensation reaction to obtain dipeptide (**F4**) in 70% yield. Next, (*N*-Me)-β-OH-Val residue precursor (**F6**) is obtained in 91% yield after a four-step reaction using chiral auxilliary method with protected sarcosine (Sar) (**F5**) as substrate [192]. The **F4** then undergoes condensation with activated **F6** to form tripeptide (**F7**) in 74% yield. The deprotected **F7** is then condensed with the protected hydroxynorvaline derivative (**F8**) [176] to produce a tetrapeptide (**F9**) in 67% yield. The deprotected **F9** is then subjected to TBDOT treatment to afford di-*N*-aminated tetrapeptide (**F10**) in 85% yield via two steps. Subsequent condensation reaction with Alloc-(*N*-OAlloc)-Ile-Cl (**F11**) [176], which is preactivated to acid chloride, affords pentapeptide (**F12**) in 67% yield. Next a three-step reaction of Mitsunobu reaction, deprotecting group and selective *N*-acylation reaction gave the hexapeptide (**F13)** in 54% yield. Finally, the hexapeptide was subjected to a four-step reaction including C/N-terminal deprotection, macrolactamization and global deprotection to obtain a pure PrgA (**F14)** in 48% yield (Scheme 10) [192].

### Cahuitamycin A

Cahuitamycin A, a non-ribosomal hexapeptide isolated from *Streptomyces gandocaensis*, and inhibited the formation of the biofilm of the human pathogenic bacterium *Acinetobacter baumannii*, which aroused chemists' interest in its synthesis [194]. Wuest and co-workers synthesized cahuitamycin A in 18% yield via 12 steps for the first time, providing a basis for future elucidation of the mechanism of the enzyme in biosynthesis [195].

The synthesis startes with 5-bromo-pentanol (**G1)** and proceeds through a seven-step reaction to obtain N2-Cbz-D-piperazinic acid (**G2)**, which is then coupled with *β*-alanine benzyl ester to obtain protected dipeptide (**G3)**. *N*_α_-Boc-*N*_δ_-Cbz-D-ornithine (**G4)** is then used as the substrate for a seven-step reaction to obtain *N*_*α*_-Fmoc-*N*_*δ*_-formyl-*N*_*δ*_-benzyloxy-D-ornithine (**G5)**. It then condenses with the dipeptide in the activated state to generate a protected tripeptide (**G6)**. The imidate is converted from nitrile (**G7)** and cyclizes with L-ser-CO_2_Bn to give **G8**, which then undergoes hydrogenolysis and coupling to produce the phenolic oxazoline fragment (**G9)**. Finally, both the deprotected **G9** and deprotected **G6** are coupled to give **G10** and then globally deprotect to give cahuitamycin A (**G11)** in 80% yield [195].

**Scheme 11:**
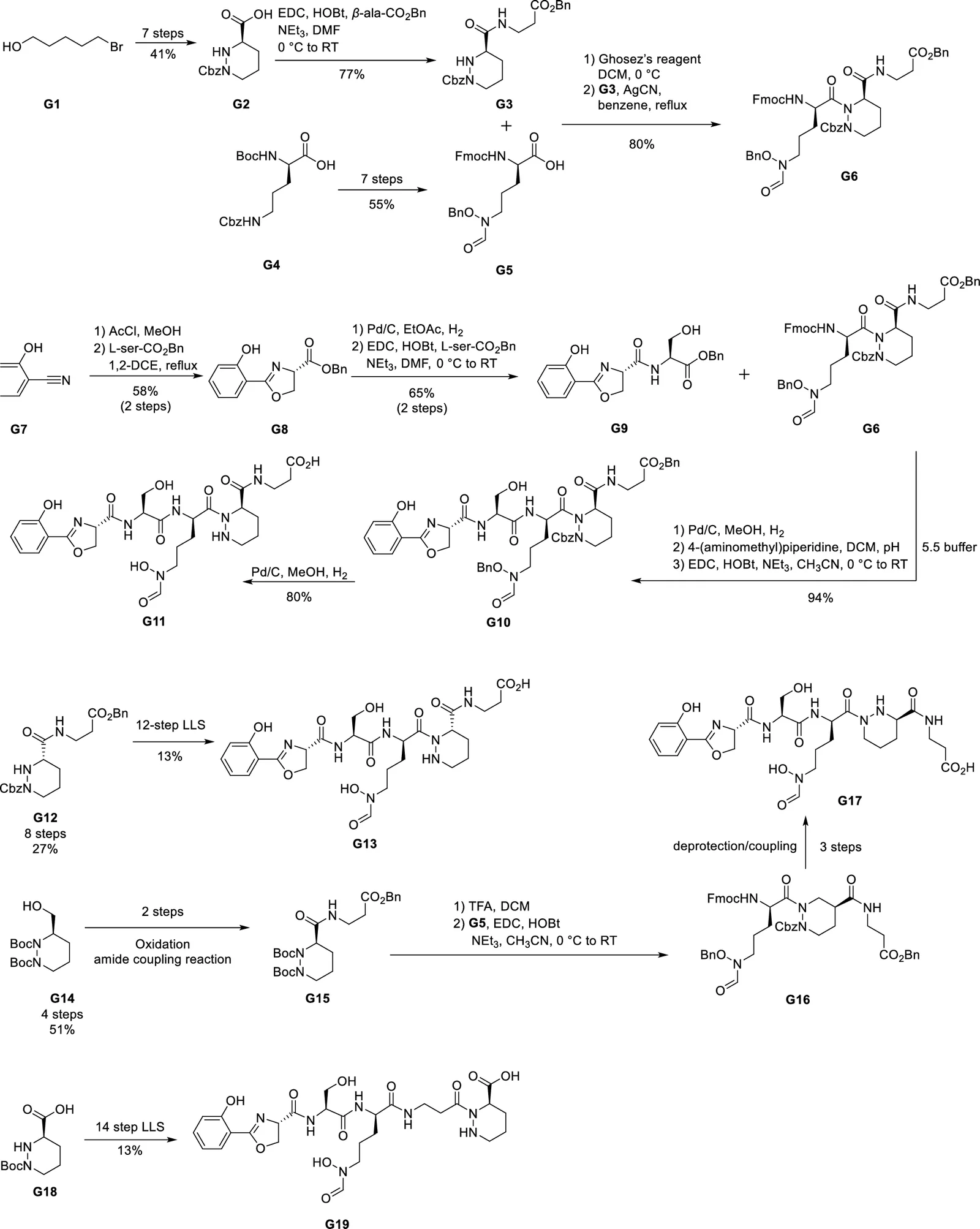
Chemical synthesis of cahuitamycin A (**G11**) and the isomer (**G17** and **G19**)

The researchers simultaneously synthesizes the isomers of cahuitamycin A. After synthesizing the enantiomeric dipeptide (**G12)** via D-proline, the epimeric isomer (**G13)** is obtained in 13% yield after a 12-step LLS with a similar deprotection/coupling sequence as before. The synthesis of the second isomer is initiated initiated by a four-step reaction to synthesize a diprotected piperazic alcohol (**G14)**, which is oxidized and amidally coupled to form **G15**. After two Boc-deprotection and three N1 position- regioselective amide coupling reactions, **G16** is obtained. The same deprotection/coupling sequence is subsequently carried out to obtain the second isomer **G17**. The *C*-terminal benzyl-protected N2-Cbz-D-piperazinic acid (**G18)** is subjected to a 14-step reaction including coupling with Fmoc-*β*-alanine followed by the deprotection/coupling sequence step to obtain the third isomer **G19** in 13% overall yield (Scheme 11) [195].

### Incarnatapeptins A and B

The synthesis of incarnatapeptins containing the piperazinic acid unit had certain difficulties. In 2024, Ye et al. performed the first total synthesis of incarnatapeptins by a retrosynthetic strategy [196].

The enone fragment is first prepared. The *N*-α-hydroxypropanoyl derivative (**H2**) in 70% yield is obtained in a two-step reaction with (*R*)-(+)-2-benzyloxypropionic acid (**H1**) as substrate, followed by diastereoselective aldol condensation to produce *anti*-aldol (**H3**) in 88% yield [197]. **H3** is then subjected to hydrolysis, esterification and oxidation to produce enone fragments (**H4**) in 95% yield (over 3 steps) [196].

Treatment with LDA and ethanal using readily available methyl (*R*)-3-hydroxybutyrate (**H5**) as the substrate produces a mixture of 1,3-*anti* diol [198] and 1,3-*syn* diol. The 1,3-*anti* diol derivative acetonide is then hydrolyzed to produce an intermediate acid (**H6**), which is then subjected to an addition reaction with the **H4** to produce a *β*-keto ester, followed by chemoselective cleavage of the acetonide and intramolecular cyclization to produce dioxabicyclo[2.2.2]octane (**H7**) in 85% yield. Subsequent deprotection affords seco acid (**H8**) in 85% yield [196].

Peptide chain synthesis is then carried out. Firstly, *N*-Cbz-L-pipecolic acid (**H9**) is subjected to Mitsunobu esterification [199, 200] to give the protected ester (**H10**) in 86% yield. Subsequent acylation with the introduced D-Piz motif (**H11**) to give **H12** which is then reacted with silver carbonate-catalyzed [201] acylation of *N*-hydroxy-L-alanine derivative (**H13**) to give tripeptide (**H14**) in 98% yield. Subsequently, the **H14** is further amidated with the introduced D-Piz subunit, and the tetrapeptide (**H15**) is obtained in 93% yield after a three-step reaction. Then β-OH-leucine (**H17**) is prepared by silylation and saponification [202] of the azide-substituted ester (**H16**) [202], and its acid chloride form is acylated with the **H15** to give the pentapeptide (**H18**) in 95% yield. Finally, hydrolysis of the protecting group and azide functional group of the pentapeptide and coupling with **H8** gave **H19**, which is then stepwise deprotected to give incarnatapeptin A (**1**) in 55% yield [196].

**Scheme 12:**
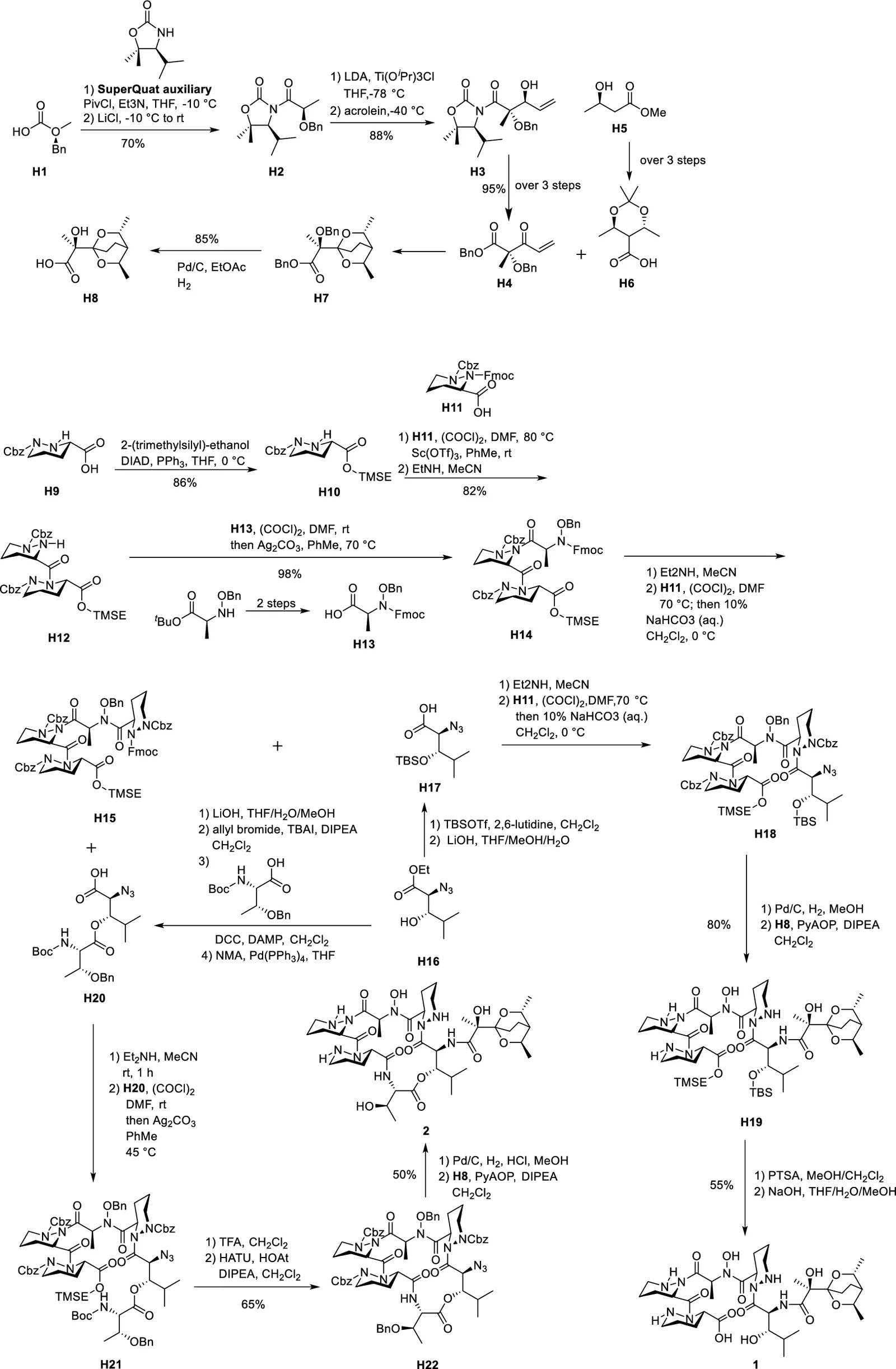
Chemical synthesis of incarnatapeptins A and B (**1** and **2**)

Incarnatapeptin B is synthesized next. The **H16** is reacted via a three-step reaction to obtain **H20**. Then *N*2 acylation with **H15** is carried out to obtain the depsipeptide framework (**H21**) in 95% yield. The macrocyclic peptide (**H22**) is then obtained in 65% yield by a two-step macrolactamization reaction. Finally, the macrocyclic peptide undergoes hydrogenolysis and acylation to produce incarnatapeptin B (**2**) in 50% yield (Scheme 12) [196].

Conventional chemical synthesis of N–N scaffolds often requires heavy metal oxidants, toxic reagents. In recent years, multiple mild, low-toxicity green synthetic strategies have been developed to address these drawbacks. First of all, by tuning reaction parameters including electrode materials, anodic oxidation enables dehydrogenative N–N coupling of amines without any transition metals, representing a green electro-synthetic strategy [203]. In addition, the enzymatic formation of biosynthetic N–N bonds is a widely used method today, offering an alternative to traditional chemical reactions that are highly polluting [203]. Moreover, multiphase recyclable catalysis enables the synthesis of compounds with high yields under ligand-free and additive-free conditions, while maintaining stable catalyst activity [204].

### Comparative analysis of synthesis strategies and bottlenecks

Among the presented tactics, the Sc(OTf)_3_-catalyzed acylation (JBIR-39) is one of the most effective methods for addressing the issue of the relatively low inherent nucleophilicity of Piz(γ-OH) derivatives. Yet its high efficiency for sterically hindered acid chlorides limits compatibility with low-bulk acyl substrates [171]. The electrophilic amination strategy enables the efficient construction of continuous piperazine acid (Piz-Piz) dipeptide backbones, overcoming the challenges posed by spatial and stereochemical factors. However, moderate coupling yields are encountered during peptide chain extension [176]. The retrosynthesis strategy is a rational approach to synthesizing large cyclic peptides. However, this method typically requires long linear sequences. For verucopeptin, the final condensation reaction to form the cyclic peptide core proceeded with a moderate yield [180], while Incarnatapeptins A and B are obtained via linear sequences as long as 12 steps, with overall yields of 26.5 and 19.7%, respectively [196].

Although ingenious methods for synthesizing cyclic peptides are currently available, several challenges remain. First, epimerization at the stereogenic center. During peptide bond formation, the α-position of piperazine acid undergoes diastereomerization [171]. Second, N-2 Acylation with Steric Hindrance. Due to the electron-withdrawing effect of the carboxyl group and the rigid conformation of the ring, the nucleophilicity of the N-2 nitrogen atom is reduced, which often renders the coupling reagent ineffective [191]. Conformation-restricted macrolactamization reactions, the cyclization of linear precursors, is often challenging and requires high-dilution conditions and potent coupling agents (Table 2) [180].

**Table 2:** Comparison of total-synthesis parameters for various natural-product compounds

Compound	Core synthetic strategy	Total steps	Overall yield
JBIR-39	Sc(OTf)_3_ catalysis	~11 steps	40%
Prolinimines A–D	Convergent biomimetic synthesis	3~4 steps	A: 36% B: 50% C: 3% D: 1%
L-156,373	Electrophilic amination	~21 steps	41%
Verucopeptin	Retrosynthesis	~20 steps	23%
Hytramycin V	Retrosynthesis	13 steps	59%
Pargamicin A	Electrophilic amination	~25 steps	48%
Cahuitamycin A	Fragment condensation	12 steps	18%
Incarnatapeptins A/B	Retrosynthesis	12 steps	A: 26.5% B: 19.7%

## Disscussion

### Conclusion

Compounds containing N–N bond functional groups are rich in variety and have good biological activities. Since the discovery of the first N–N bond-containing natural product macrozamin, more and more N–N bond-containing compounds, especially those of microbial origin, have been the subject of avid research by chemists. This review focuses on the isolation, biological activities, key mechanisms of N–N bond formation, synthesis and biosynthesis, and pharmacological aspects of N–N bond-containing NPs of microbial origin since 2014, which are comprehensively summarized and outlined. Currently, 131 types of N–N bond-containing NPs are summarized from 2014 to 2024 (Table 3), of which, mainly actinomycetes as the main source and fungal sources accounted for a few.

**Table 3:** Structures and associated biological evaluations of Nitrogen−Nitrogen Bond-Containing Natural Products

No	Compound name	Strains	Source	Activities	References
*Hydrazides* (**58**)					
1	Incarnatapeptin A (**1**)	*Streptomyces incarnatus* NRRL 8089	Soil (Indian soil)	–	[20]
2	Incarnatapeptin B (**2**)	*Streptomyces incarnatus* NRRL 8089	Soil (Indian soil)	Anti-prostate cancer	[20]
3	Meliponamycin A (**3**)	*Streptomyces* sp. ICBG1318	Animal (*Melipona scutellaris* nurse bees)	Antimicrobial	[29]
4	Meliponamycin B (**4**)	*Streptomyces* sp. ICBG1318	Animal (*Melipona scutellaris* nurse bees)	Antimicrobial, antiprotozoal	[29]
5	Albofungin A (**5**)	*Streptomyces chrestomyceticus*	Other	Antimicrobial, anticancer	[30]
6	JBIR-39 and JBIR-40 homolog 6 (**6**)	Endophytic *Streptomyces* sp. AB100	Plant (*Atropa belladonna* (L.) shoots)	Antimicrobial	[31]
7	JBIR-39 and JBIR-40 homolog 7–8 (**7**–**8**); JBIR-39 and JBIR-40 homolog 10–11 (**10–11**)	Endophytic *Streptomyces* sp. AB100	Plant (*Atropa belladonna* (L.) shoots)	–	[31]
8	JBIR-39 and JBIR-40 homolog 9 (**9**)	Endophytic *Streptomyces* sp. AB100	Plant *Atropa belladonna* (L.) shoots	Antimicrobial	[31]
9	Dentigerumycins F–G (**12**–**13**)	*Streptomyces incarnatus* NRRL 8089	Other	–	[21]
10	Petrichorins A–B (**14**–**15**)	*Lentzea flaviverrucosa* DSM 44664	Other	Anticancer	[32]
11	Polyoxyperuin A seco acid (**16**)	*Streptomyces* sp. s120	Marine (peruvian coast)	Antibacterial	[33]
12	Lydiamycin E (**17**)	*Streptomyces* sp. s120	Plant (leaves of *Cinnamomum cassia*)	–	[34]
13	Lydiamycins F–H (**18**–**20**)	*Streptomyces* sp. s120	Plant (leaves of *Cinnamomum cassia*)	Anti-migratory activity against PANC-1 cells	[34]
14	*N-*aminoanthranilic acid (**21**)	*Penicillium paneum* SD-44	Marine (marine sediment)	–	[36]
15	PM130391 (**22**); PM130392 (**23**); PM140293 (**24**)	*Streptomyces tuirus*	Marine (marine sediment)	Anticancer	[37]
16	Lunaemycins A–B1 (**25**–**26**); lunaemycin D (**30**)	*Streptomyces lunaelactis* MM109T and MM37	Mineral deposits (moonmilk deposits)	Antibacterial	[24, 38–40]
17	Lunaemycins B2–C2 (**27**–**29**); lunaemycins E1–J3 (**31**–**39**)	*Streptomyces lunaelactis* MM109T and MM37	Mineral deposits (moonmilk deposits)	–	[24,^38^–^40^]
18	Lenziamide D1 (**40**); lenziamide B1 (**41**)	*Streptomyces* sp. S063 CGMCC 14582	Marine	Anticancer	[41]
19	Polyoxyperuin B seco acid (**42**)	*Streptomyces* sp. GB16	Other	–	[8]
20	Depsidomycin D (**43**)	*Streptomyces* sp. BYK1239	Other	–	[8]
21	Lenziamide A (**44**)	*Streptomyces* sp. PC5	Animal (dung beetle *Onthophagus lenzii*)	Anticancer	[8]
22	Lenziamide B (**45**)	*Streptomyces* sp. SNJ018	Marine sediment	Anticancer	[8]
23	Caveamide A (**46**)	*Streptomyces* sp. BE230	Mineral deposits (New Rankin Cave)	Anticancer	[43]
24	Caveamide B (**47**)	*Streptomyces* sp. BE230	Mineral deposits (New Rankin Cave)	Anticancer, antimicrobial	[43]
25	Diperamycin (**48**)	*Streptomyces* sp.	Animal (cuticle of leaf-cutting ants of the tribe *Attini*)	–	[44]
26	Depsidomycins B (**49**) and C (**50**)	*Streptomyces* sp. BYK1371	Ginseng farm soil	Anti-metastatic activity in metastatic breast cancer	[46]
27	Hydrazidomycin D (**51**)	*Streptomyces* sp. RKBH-B178 and *Mycobacterium smegmatis*	Other	Anticancer	[26]
28	Svetamycin A (**52**); svetamycin C (**54**); svetamycins F–G (**57–58**)	*Streptomyces* sp.	Other	Antimicrobial	[47]
29	Svetamycin B (**53**); svetamycins D–E (**55–56**)	*Streptomyces* sp.	Other	–	[47]
*Hydrazines* (**4**)					
30	Peniproline A (**59**)	*Penicillium decumbens* CP-4	Plant (*Cymbidium mannii* Hook. f.)	Anticancer	[48]
31	FST R (**60**)	*Streptomyces albus* J1074	Marine (marine sediment)	–	[49]
32	Difluostatin B (**61**)	*Streptomyces albus* J1074	Marine (marine sediment)	–	[49]
33	amycophthalazinone A (**62**)	*Amycolatopsis* sp. YIM 130642	Plant (lichen *Squamarina* sp.)	Antimicrobial	[50]
*Hydrazones* (**19**)					
34	Farylhydrazone C (**63**)	*Penicillium* sp. HDN14-431	Soil	Antimicrobial	[51]
35	s56-p1 (**64**)	*Streptomyces lividans* TK23	Other	–	[52]
32	Prolinimines A–D (**65**–**68**)	*Trichoderma* sp. CMB-F563	Animal (fish gastrointestinal tract)	–	[55]
33	Murayaquinone C (**69**)	*Streptomyces* sp. NA4286	Animal (insect)	–	[56]
34	Penoxahydrazone A (**70**)	*Penicillium oxalicum*	Marine (deep sea cold seep)	Anti-microalgae, antimicrobial, low toxicity against marine zooplankton	[57]
35	Penoxahydrazones B–C (**71**–**72**)	*Penicillium oxalicum*	Marine (deep sea cold seep)	–	[57]
36	Tasikamides A–C (**73**–**75**)	*Streptomyces tasikensis* P46	Quarry lake sediment	–	[58]
37	Penzonemycin A (**76**)	*Streptomyces* sp. SCSIO 40020	Marine (Pearl River Estuary sediment)	Anticancer	[59]
38	Penzonemycin B (**77**)	*Streptomyces* sp. SCSIO 40020	Marine (Pearl River Estuary sediment)	–	[59]
39	Talarohydrazone A (**78**)	*Talaromyces amestolkiae* HDN21-0307	Marine (deep-sea cold seep)	Anticancer	[22]
40	Talarohydrazones B–D (**79–81**)	*Talaromyces amestolkiae* HDN21-0307	Marine (deep-sea cold seep)	–	[22]
*Azo and azoxy* (**21**)					
41	KA57A (**82**)	*Streptomyces rochei*	Other	–	[61]
42	Azoxymycins A–C (**83–85**)	*Streptomyces chattanoogensis* L10	Other	–	[62]
43	Azodyrecin A (**86**)	*Streptomyces* sp. P8-A2	Soil (Denmark soil)	Brine shrimp toxicity	[63]
44	Azodyrecin B (**87**)	*Streptomyces* sp. P8-A2	Soil (Denmark soil)	Anticancer	[63]
45	Azodyrecin C (**88**)	*Streptomyces* sp. P8-A2	Soil (Denmark soil)	–	[63]
46	Azodyrecins D–G (**89**–**92**)	*Streptomyces* sp. RM72 and *Streptomyces* sp. A1C6	–	–	[64]
47	*O*-Alkylazoxymycin A (**93**);* O*-Alkylazoxymycins E–F (**97–98**)	*Streptomyces* sp. Py50	Plant (*Pyrrosia lingua*)	Antimicrobial	[65]
48	*O*-Alkylazoxymycins B–D (**94–96**)	*Streptomyces* sp. Py50	Plant (*Pyrrosia lingua*)	–	[65]
49	Maniwamycins C–F (**99–102**)	*Streptomyces* sp. TOHO-M025	Soil (Japan)	Quorum-sensing inhibitors; antimicrobial	[66]
*Nitrosohydroxylamines* (**15**)					
50	Gramibactin (**103**)	*Paraburkholderia graminis*	Soil (rhizosphere and disease-suppressing soils of an agricultural sugar-beet field)	Siderophore	[67]
51	Gramibactin B (**104**)	*Paraburkholderia caledonica*	Soil (rhizosphere and disease-suppressing soils of an agricultural sugar-beet field)	Siderophore	[67]
52	Megapolibactins A–F (**105–110**); Megapolibactin A_cyc_ (**111**)_;_ Megapolibactin B_cyc_ (**112**)	*Paraburkholderia megapolitana*	Plant (moss)	Siderophore	[67]
53	Plantaribactin (**113**)	*Burkholderia plantarii*	Plant	Siderophore	[67]
54	Gladiobactin (**114**)	*Burkholderia gladioli*	Plant	–	[67]
55	Trinickiabactin (**115**)	*Trinickia caryophylli* DSM 50341	Plant	Antimicrobial	[68]
56	Tistrellabactins A–B (**116**–**117**)	*Tistrella mobilis* KA081020-065	Marine (Red Sea)	Siderophore	[69]
*Nitrogen-containing heterocycles* (**14**)					
57	WS9326H (**118**)	*Streptomyces* sp. SNM55	Soil (mudflat)	Antiangiogenic	[71]
58	Difluostatin I (**119**)	*Micromonospora rosaria* SCSIO N160	Marine (South China Sea)	–	[72]
59	Nocuolactylate A (**120**)	*Nodularia* sp. LEGE 06071	Other	Anticancer, antimicrobial	[73]
60	Nocuolactylate B (**121**)	*Nodularia* sp. LEGE 06071	Other	Anticancer	[73]
61	Nocuolactylates C–E (**122**–**124**)	*Nodularia* sp. LEGE 06071	Other	–	[73]
62	Chaetoglobosin B1 (**125**)	*Chaetomium madrasense* 375	Soil (China)	Anticancer	[74]
63	Chaetoglobosins B2–B3 (**126–127**)	*Chaetomium madrasense* 375	Soil (China)	–	[74]
64	pyrazolofluostatin A (**128**)	*Micromonospora rosaria* SCSIO N160	Marine (South China Sea)	Antioxidation activity	[75]
65	pyrazolofluostatins B–C (**129–130**)	*Micromonospora rosaria* SCSIO N160	Marine (South China Sea)	–	[75]
66	A phenyl pyridazine derivative (**131**)	*Aspergillus* sp. AV-2	Mangrove forests	–	[76]

“–” means no activity

This review primarily discusses the structural diversity, biosynthesis, chemical synthesis, and pharmacology of N–N-bond-containing compounds. These aspects lay the foundation for future pharmacological research on such compounds. In terms of strain origin, plant-derived strains accounted for the highest proportion (22.1%), indicating that strains isolated from plants are an important source of these metabolites. Furthermore, compounds containing N–N bonds exhibit a wide range of activity, primarily including anticancer, antimicrobial, anti-microalgae, siderophore. This indicates that N–N bond-containing natural products hold significant potential in anticancer and anti-infective drug discovery.

**Fig. 10:**
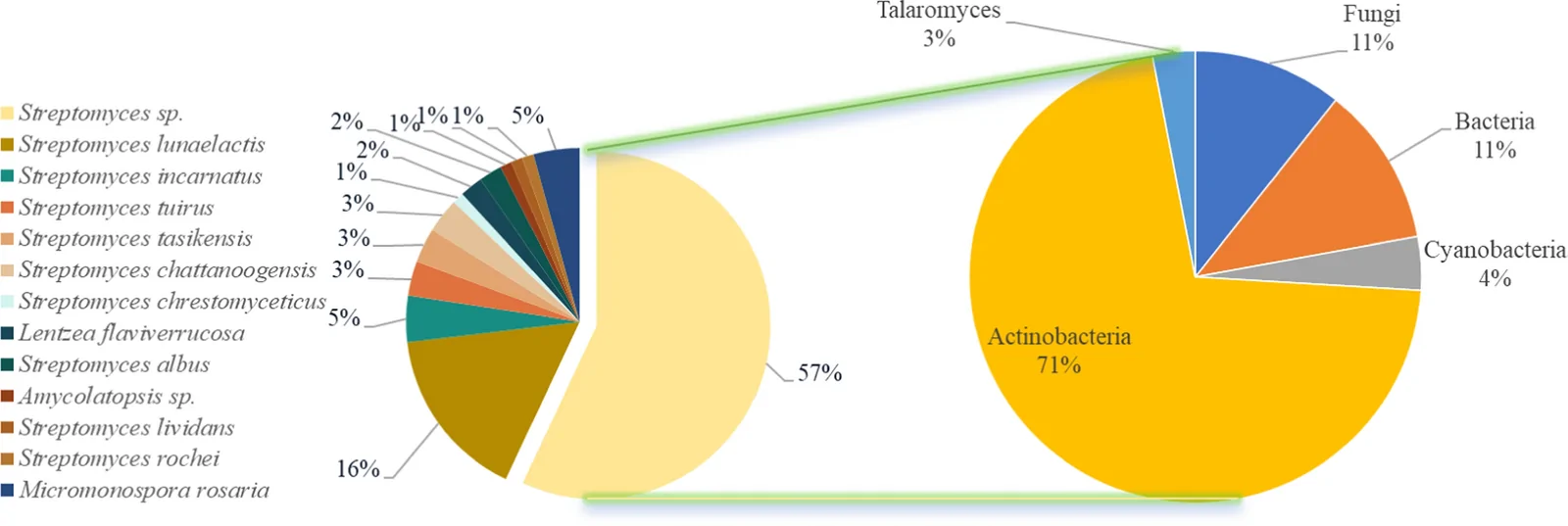
Microbial source distribution of 131 N–N natural products, 2014–2024

**Fig. 11:**
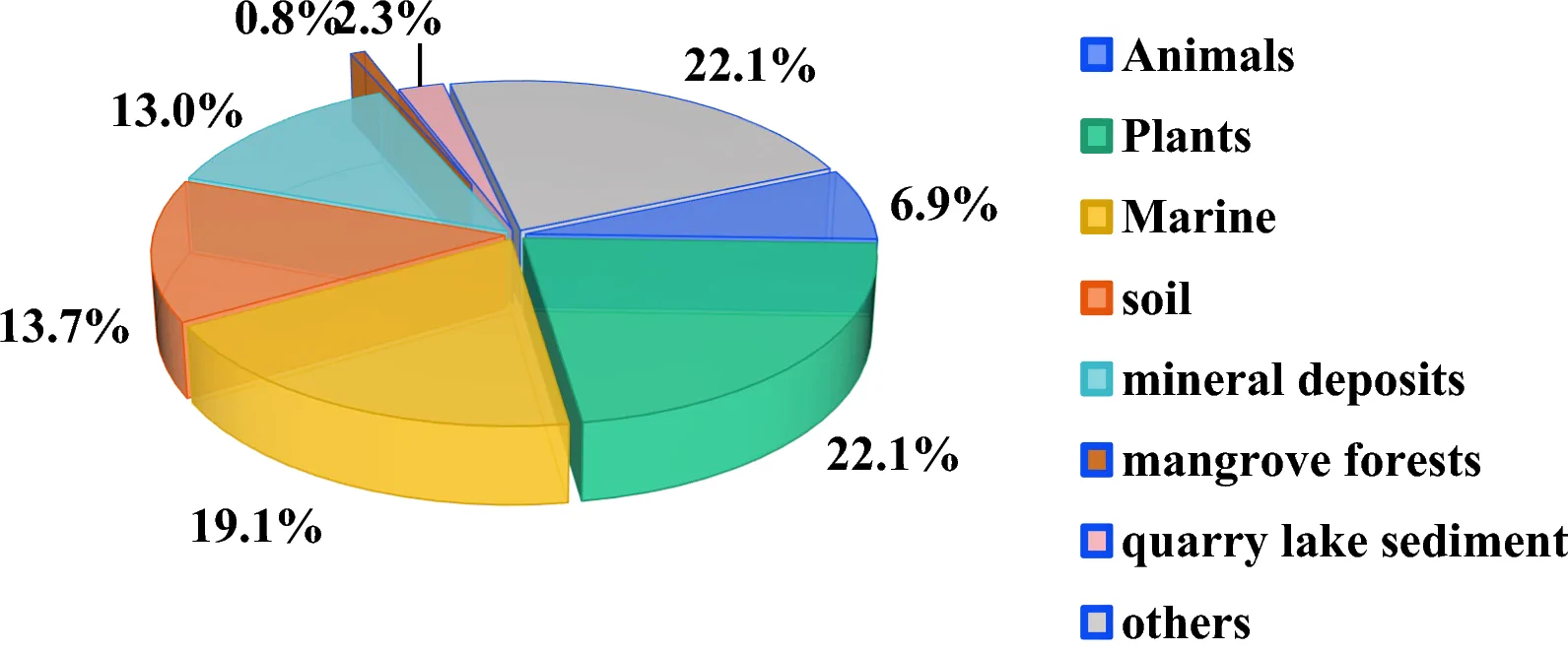
Habitat distribution of microbial strains producing N–N natural products, 2014–2024

In addition, among microbial sources, those derived from actinobacteria account for 93 compounds (71%) which strongly supports the central role of actinomycetes (especially *Streptomyces*) as the most prolific producers of N–N bond-containing natural products. These *Streptomyces* strains originate from diverse ecological niches, including soil, deposits, marine sediments et al. From the perspective of biosynthetic gene clusters, three key advantages make *Streptomyces* the primary producer of N–N-bond-containing natural products. First, *Streptomyces* genomes harbor a large number of silenced NRPS biosynthetic gene clusters. However, through methods such as cloning and reconstruction of BGCs (applied in heterologous hosts), engineering of promoters, regulatory factors, and ribosomes (applied in native hosts), or approaches like OSMAC, latent N–N biosynthetic pathways can be activated, thereby yielding a large number of structurally novel compounds [205]. Second, *Streptomyces* possesses multiple manipulating regulators that can produce compounds such as piperazine acid through the conserved ktzI/ktzT gene cassette, which is widely distributed within these biosynthetic gene clusters [206]. Third, their massive genomic sequences (7–12 Mb) contain a large number of enzyme genes (cytochrome P450, acetyltransferases) that are used to diversely modify skeletons containing N–N bonds (Figs. 10 and 11) [207, 208].

According to functional group classification, these compounds are classified into six categories based on their functional groups. Pyrazinic acid derivatives are primarily isolated from the *Streptomyces*, among these, compounds **5** [30], **14**–**15** [32], **18**–**20** [34] exhibit significant antitumor activity. Hydrazines are primarily isolated from the genus *Penicillium*, *Streptomyces*, *Amycolatopsis.* Hydrazones are primarily isolated from the genus *Streptomyces*, among these, compound **70** [57] has a relatively unique structure and exhibit good inhibitory activity against microalgae and marine pathogenic bacteria. Azo and Azoxy are primarily isolated from the genus *Streptomyces*, among these, compound **87** [63] exhibit cytotoxicity. Nitrosohydroxylamines are primarily isolated from the genus *Paraburkholderia*, among these, trinickiabactin exhibit strong antimicrobial activity. Nitrogen-containing heterocycles are primarily isolated from the genus *Streptomyces*, *Micromonospora*, *Nodularia*, among these, compound **118** [71] exhibit antiangiogenic activity, laying the groundwork for future in-depth pharmacological research.

The first step in biosynthesis is to solve the problem of N–N bond synthesis. Several pathways and synthetic methods for N–N bond formation in the functional groups of such natural products have been identified, mainly including the nitrite ANS pathway, hydrazine synthase, radical coupling and NO-dependent pathway. The complete biosynthetic pathways of many N–N-type natural products have not yet been fully elucidated. Since several analogs share the same conserved biosynthetic mechanisms as characterized compounds, further enzymatic studies will shed light on these unknowns [17]. Chemical synthesis of N–N bond-containing NPs is also a direction of interest, and natural products prepared by chemical total synthesis are more advantageous in the preparation of chemicals. Currently, several synthetic strategies exist for the total synthesis of N–N bond-containing NPs, primarily including Sc(OTf)_3_ catalysis, electrophilic amination, and retrosynthesis. These synthetic strategies also provide new ideas and references for other compounds.

**Fig. 12:**
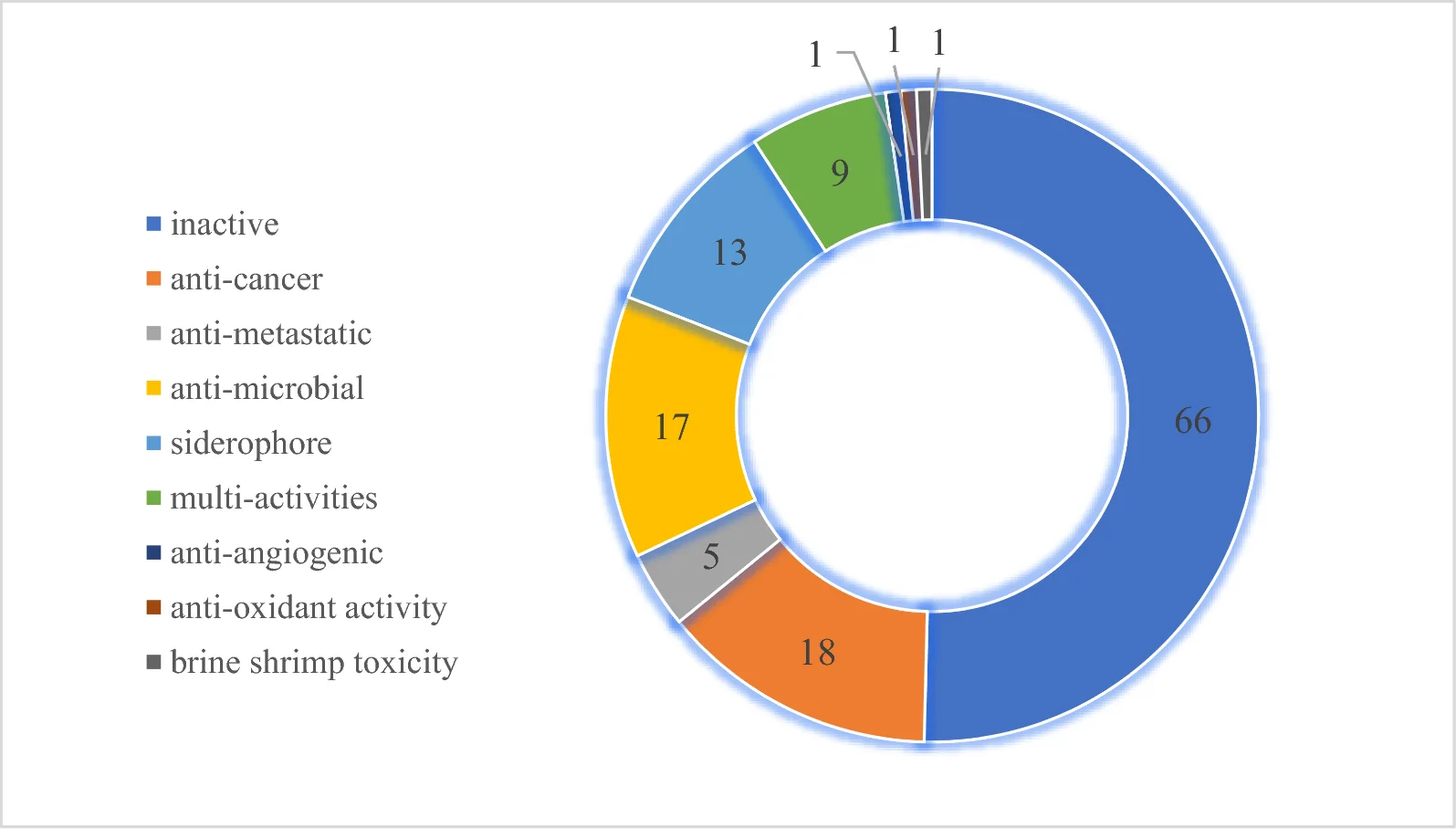
Bioactivity classification of 131 N–N natural products, 2014–2024

**Fig. 13:**
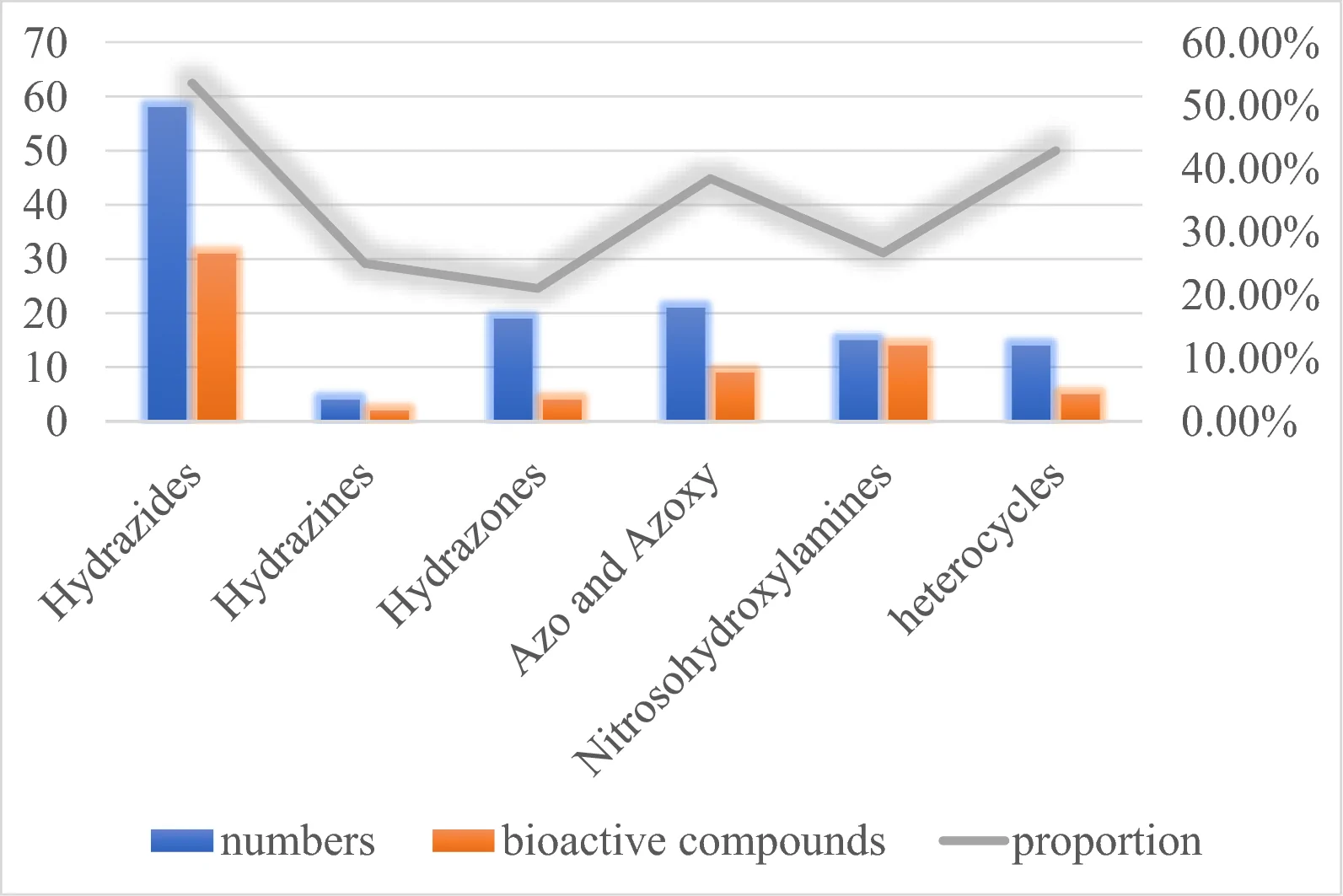
Quantitative characteristics and bioactivity distribution of different structural types of 131 N–N natural products, 2014–2024

As shown in Fig. 12, the proportion of inactive molecules varies across different structural subgroups of natural products containing N–N bonds, with inactive compounds accounting for 50.38% of the total. This may be due to differences in activity resulting from structural variations. It is worth noting that, among all structural subgroups, derivatives containing piperazine acid exhibit a relatively high anticancer hit rate. As discussed in Sect. 3.2, the saturated N–N single bond in piperazine acid, combined with its moderate electron-donating ability and rigid ring conformation, can interact synergistically with adjacent peptide residues, thereby forming a specific binding pocket for tumor-associated proteins. This explains why most compounds containing piperazine acid generally exhibit good antitumor activity (Figs. 12 and 13).

Although the biological activity of N–N bond-containing natural products is strong, there is a lack of relevant experimental data in pharmacological studies. To date, only verucopeptin has been fully characterized, which reverses cancer multidrug resistance by targeting ATP6V1G1 and inhibiting mTORC1 [80]. Most analogs exhibit only crude cytotoxic, antibacterial, or anti-angiogenic activity and research into the target mechanisms of these molecules will also play a significant role in future drug development.

### Outlook

Based on the above comprehensive summary, there are still some issues with compounds containing N–N bonds that need to be addressed in the future in order to advance research on natural products containing N–N bonds.

First, mining silent biosynthetic gene clusters via OSMAC/GNPS. Although actinomycetes (particularly Streptomyces) are the most prolific producers of N–N bonds-containing compounds, their genomes contain a large number of silenced biosynthetic gene clusters (BGCs) that cannot be normally expressed under experimental conditions. To uncover this hidden chemical diversity, the following strategies should be adopted: (i) the OSMAC approach, which activates gene clusters by adjusting fermentation parameters. (ii) GNPS-based molecular network analysis, which links compounds with similar clusters by comparing MS/MS fragmentation spectra, enabling rapid identification of compounds containing N–N bonds. (iii) The host strain is optimized by modifying the promoter construct and using heterologous expression targeting BGCs, thereby increasing the yield [49].

Second, Develop simple and environmentally friendly synthetic routes. As discussed in Sect. 6.9, the synthesis of cyclic peptides remains hampered by low yields and challenging macrocyclization reactions. Future research should prioritize: (i) optimizing the reactivity of piperazine acid to avoid the formation of isomeric byproducts; (ii) exploring more environmentally friendly alternatives, such as biosynthetic enzymes, electrochemical N–N coupling, and recyclable catalysis.

Third, Utilizing proteomics and molecular docking to clarify pharmacological targets and SAR. For most N–N bond-containing compounds, molecular targets and SAR remain unknown. Future studies should employ: (i) Proteomics and molecular docking techniques should be utilized to further refine the study of the molecular mechanisms underlying N–N bond-based pharmacophores in the future [80]; (ii) Currently, the SAR of most natural products containing N–N bonds have not yet been studied, and there are significant differences in activity between the distinct structures of caveamides A and B [43]. Conducting systematic SAR studies through appropriate chemical modifications of key pharmacophores is of great significance for identifying target proteins for future drug molecules.

## Data Availability

The original contributions presented in the study are included in the article/Supplementary Material; further inquiries can be directed to the corresponding author.
